# Detecting Square Grid Structure in an Animal Neuronal Network

**DOI:** 10.3390/neurosci3010007

**Published:** 2022-02-03

**Authors:** Robert Friedman

**Affiliations:** Retired from Department of Biological Sciences, University of South Carolina, Columbia, SC 29208, USA

**Keywords:** neural system, fruit fly brain, neuronal network, square grid arrangement, network metrics

## Abstract

An animal neural system ranges from a cluster of a few neurons to a brain of billions. At the lower range, it is possible to test each neuron for its role across a set of environmental conditions. However, the higher range requires another approach. One method is to disentangle the organization of the neuronal network. In the case of the entorhinal cortex in a rodent, a set of neuronal cells involved in spatial location activate in a regular grid-like arrangement. Therefore, it is of interest to develop methods to find these kinds of patterns in a neural network. For this study, a square grid arrangement of neurons is quantified by network metrics and then applied for identification of square grid structure in areas of the fruit fly brain. The results show several regions with contiguous clusters of square grid arrangements in the neural network, supportive of specialization in the information processing of the system.

## 1. Introduction

The animal neural network is often generalized as a model of a small-world arrangement of neurons and their connections [1]. A departure from this network model includes regular shapes and arrangements formed between interconnected neurons, such as the case of a lattice-like structure. A lattice is commonly defined as a regularity of structure in a formal graph, such as a hexagonal, rectangular, or triangular grid arrangement, but the precise definition of lattice varies across studies [2,3]. In this study, the lattice detection algorithm is restricted to a search for a square grid-like pattern, similar to that observed in the grid cells which fire in a hexagonal arrangement in the medial entorhinal cortex of a rodent [4,5,6,7].

**Figure 1:**
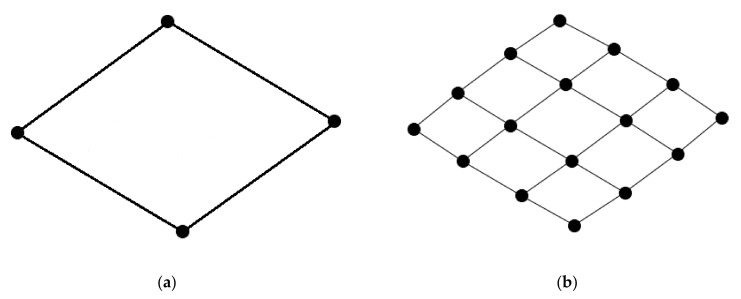
Images of square grid arrangements. (**a**) Single square grid defined as a cycle graph of four nodes; (**b**) a lattice-like structure that is represented by a regularly repeated pattern of square grids.

A square grid, equivalent to a rectangular grid, is the arrangement of interest, particularly since it is differentiable from the common triangular grid pattern of a small-world neuronal network. The minimal square grid arrangement is defined as a cycle graph of four nodes (C_4_; Figure 1a). This arrangement may be repeated across a network and may be referred to as a lattice-like structure of square grids (Figure 1b).

Network metrics that search for triangular grids, such as transitivity, are not applicable by themselves for identifying these square grid patterns. Likewise, there are other related metrics that are not applicable by themselves to finding grid-like patterns. An example is a metric for finding bipartitions in a network, spectral-based bipartivity, and it is relevant for identifying both grid-like and star-like network arrangements [8]. The star-like network structure does not share a shape or pattern with a regular grid. Therefore, other metrics are required to provide differentiation in isolating the pattern of interest.

Furthermore, a methodology for measuring idealized network patterns is not without assumptions. One assumption here is that the lattice-like structure contains a neuron at each vertex of a regular grid. However, the patterns in an animal neuronal network may consist of populations of neurons at the vertices, a confounding factor in searching for the patterns, and which may lead to departures from detection of the idealized pattern of interest [9]. In this study, with an emphasis on non-conservative search criteria, it is possible to detect square grids in cases that are departures from an idealized form of a grid.

In addition, the regular grid structure represents a localization of information [5,10], as exemplified by a model of particles traveling across a network, and correspondingly their level of efficiency and capability in routing [11]. Empirical studies support this model, such as in the dynamics of neuronal cells in tissue culture which show a dependence on network topology, a result accompanied by replication in machine simulation [12,13].

The fruit fly hemibrain project [14] released data on a portion of the central brain of the female adult fly. It includes information on the neurons and their connections. In this study, there is a search for the square grid pattern in this data set. Simulated data was also generated to create an informatic-driven approach for identification of these grids. In general, regular patterns in neuronal networks are of interest because they have been observed in the activation of grid cells, and furthermore, information theory predicts that these kinds of patterns are associated with specific dynamics in the processing of information [11].

## 2. Methods

### 2.1. Simulating Neural Network Structure

**Figure 2:**
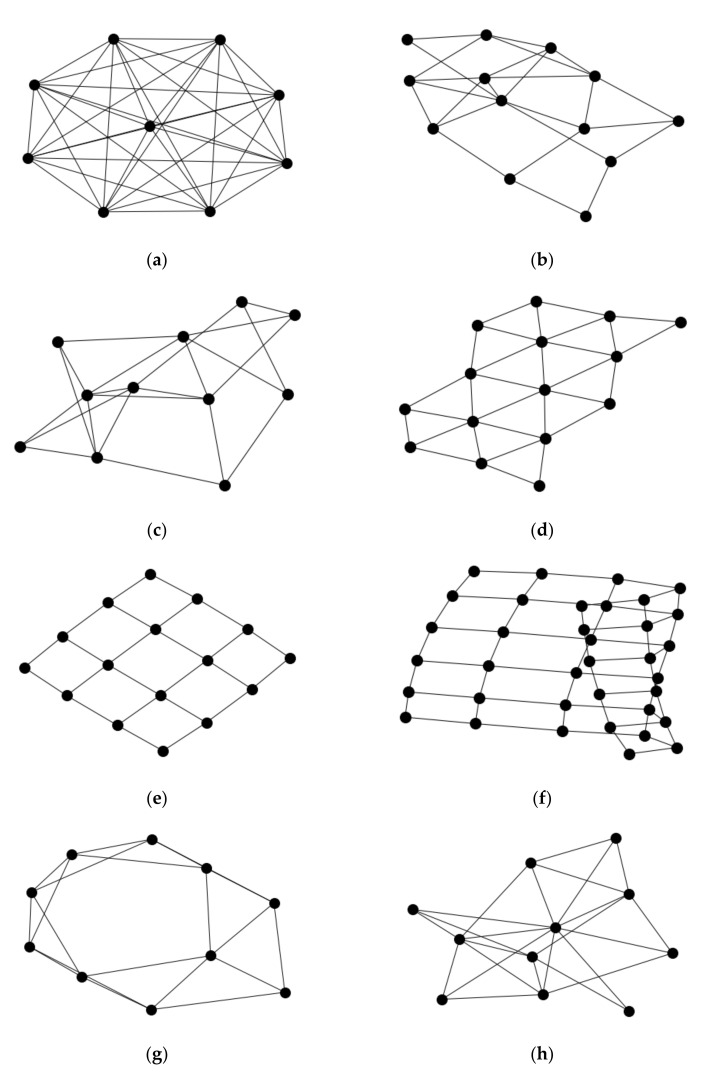
Images of simulated networks by the model of generation and drawing orientation. (**a**) Complete network; all nodes of the network share an edge. (**b**) Random network; example of a network where the edges are randomly added between pairs of nodes. (**c**) A second example of a random network. (**d**) Triangular grid network. (**e**) Square grid network. (**f**) Square grid network where a portion of the network is folded upon itself. (**g**) Small-world network. Edges added to network prefer to attach to nodes that are neighbors. (**h**) Preferential attachment network. Edges added to network prefer to attach to nodes that are already attached to other nodes.

Five models were applied for generating neuronal networks, along with a subsequent analysis to collate a set of metric values that describe the network topology and its properties. These network models include the complete graph (Figure 2a), a random network (Figure 2b), a lattice grid-like network (Figure 2d,e), a network with preferential attachment [15] (Figure 2h), and a network with a small-world architecture [2] (Figure 2g). These are parameterized models. Parameters include the number of nodes in the above models, while the models for preferential attachment and small-world include additional parameters for adding new connections. The simulations were repeated for each model so that the networks span a range of parameter values. However, these parameters are often not applicable to small samples of interconnected nodes. The parameter values were chosen to reflect this constraint. Moreover, in general, these networks are better described as a network component since this is the unit of computational analysis, and the related metrics are often applicable to the presence of a contiguous network.

The Supplementary Materials contain the python code, version 3, to generate the simulation data, and the results for the 2d lattice simulations. The python library, NetworkX, provides the code and implementation for calculation of these network metrics [16].

Metric values for detecting square grids include the square clustering coefficient [17], average shortest path length in the network, spectral bipartivity [8], transitivity, and the small-world coefficient sigma [18]. The square clustering coefficient is for detection of squares of nodes, a cycle of four connections, which form between a subset of nodes. This metric is a verifier for the bipartivity measurement.

The bipartivity detects bipartitions in a network where the network is separable into two components. The bipartivity metric associates a square grid arrangement with its highest value, 1.0, but it also detects a star-like network with high values. This confounding factor is an example of a reason to use multiple metrics in the search method, and the addition of other metrics is expected to increase precision in network pattern detection and provide verification for positive identification of a square grid arrangement.

These metric values for square grid detection are applied in a procedure for searching the neuronal network data of the fruit fly brain [14]. This part of the study serves as a verifier on the robustness of estimates of square grid-ness from the network simulations, and likewise, this neuronal data will lead to the reconstruction of network patterns that are not available to a machine simulation, such as the patterns found in hybrid models. For this case, the false positive patterns are observable, a result of a false identification of a square grid pattern. However, the true negative rate is not observed, but this kind of error is minimized by the use of many metrics in the search procedure.

Omega [3] is an alternate metric for stringently detecting small-world architecture. This metric is based on the lattice definition by Watts and Strogatz [2]. However, the omega algorithm is dependent on the transitivity metric, and therefore, a triangular grid pattern. For this reason, omega is excluded from the search criteria for a square grid arrangement, as it is not applicable to square grid architecture.

### 2.2. Fruit Fly Hemibrain Data

The neuron and synapse data are available in an archive from the fruit fly hemibrain project [14] (version 1.1, 21 June 2020, neuprint.janelia.org). The data set of interest has the interconnected neurons that are assigned to a region of interest (ROI) [19]. These regions were originally classified by distinctiveness in the synaptic density of nerve fiber, which occurs within and between regions across an animal brain. The brain regions of this data set are abbreviated by a suffix if associated with the left or right hemisphere (L/R). The data set includes 61 files which map to an ROI, each representative of a different region or hemisphere in the central portion of the fly brain. Moreover, an additional data file references neurons unmapped to an ROI, and includes 35,022 records, where each record is a connected pair of neurons.

These brain regions, and their abbreviations, are also anatomically classified by membership within a synapse-rich neuropil [19] (Table 1).

**Figure 3:**
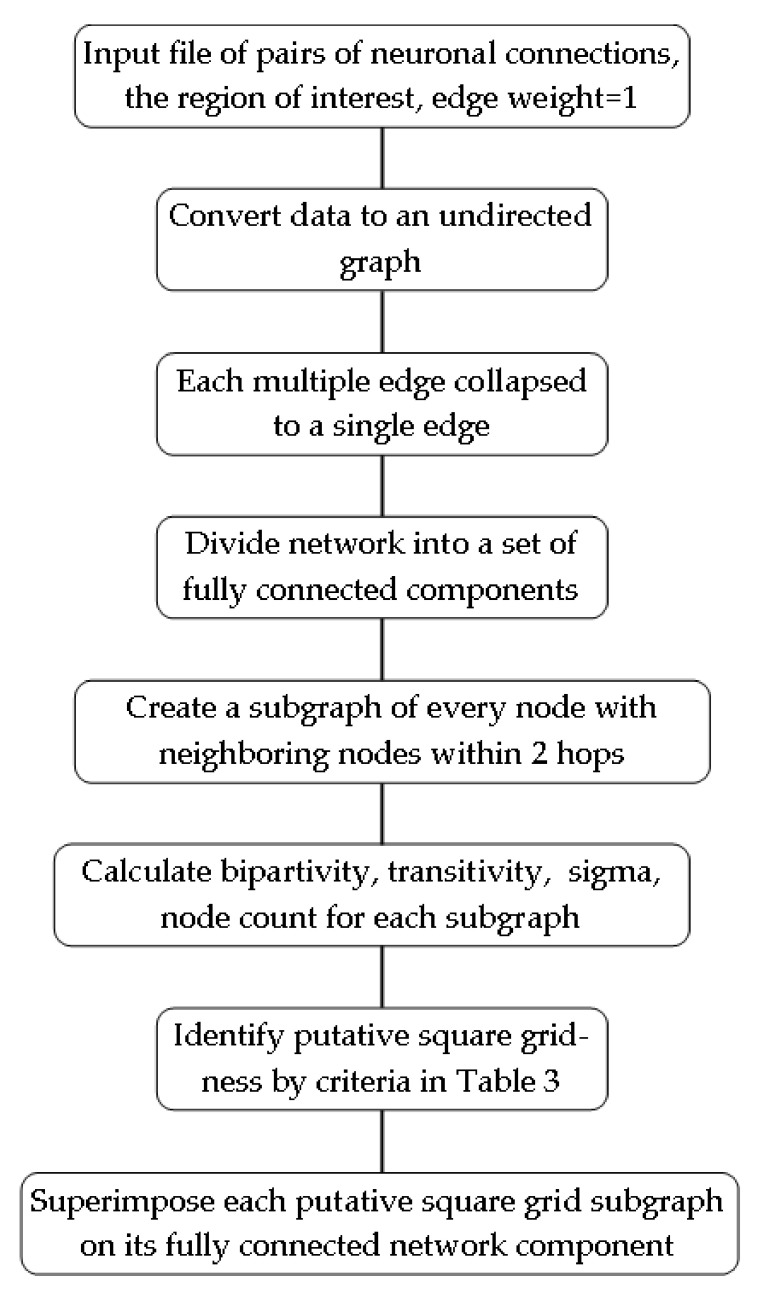
Process for identifying square grid-ness in a network based on neuronal connection data.

The hemibrain data file is in text format. It contains records for over 20,000 neurons, along with their connections by region of interest. The R code, in the Supplementary Materials, converts this data file for computational analysis (see Figure 3 for workflow).

### 2.3. Analysis of the Hemibrain Data

The hemibrain data project [14], applicable to a portion of the central brain of the fruit fly, was analyzed for identification of square grid structure (Figure 2e) in the neuronal network. The simulation data provided the framework to find parameters and their values that correspond to square grid-ness, while excluding non-square grid arrangements as generated by the simulated network models, such as in the random and preferential attachment graphs as described earlier [16]. However, there is not a single metric that specifically matches a network arrangement of a square grid pattern, so this study required use of many parameters in detecting square grid arrangements.

The detection of false positive matches is undertaken by steps in this square grid-ness detection procedure (Figure 3). First, detections must correspond to a sufficiently large sample of neurons. This is accomplished by searching for square grids in a sliding window of nodes along the neural network. Each window includes the node of interest, its adjacent neighbors, and also all adjacent neighbors of those neighbors. Therefore, each node of interest forms a set of nodes which includes all neighbors within two steps away, and this defines the window size for sampling the neuronal network.

All nodes are involved in this sampling process across the network, and the subnetworks that correspond to the windows are assigned square grid-ness scores by use of the specified metrics. Each window is also inspected by eye as a final verification step, but more importantly, to verify the occurrence of overlap between windows that are classified as square grid-like. The overlapping of these windows leads to a larger square grid-like structure, fitting with an expectation of a functionally significant arrangement.

The above windows, the units of data analysis, are displayed as network images and archived as files in the Supplementary Materials. These files show the results of the window analysis as described above. The search for overlapping windows, the larger square grid-like structure, is described in the results section.

The relevant parameters for the search of square grid-ness included transitivity, bipartivity, and sigma. Transitivity is a metric for the ratio of the number of triangles to the total possible number of triangles in a network. A possible triangle is counted by number of triads, where a triad is two edges of a graph with a shared vertex.

### 2.4. Computational Resources

The adult fruit fly hemibrain data [14] was analyzed by executing python code that includes access to network metrics from the NetworkX library [16]. For the computational work, Google Colaboratory [20] provided a web-based service with a Jupyter Notebook platform [21], a development system for coding and debugging, along with libraries of mathematical functions, such as NetworkX. This platform was used for testing and running the python code, included in the Supplementary Materials, for analysis of the hemibrain data and simulation of the networks. Another advantage of Colaboratory is that code is remotely run on remote computer processors.

In the case here, NetworkX is a single-threaded library, along with the python code interpreter that is single-threaded in its operations. The remote computer processing unit, classified as a Haswell, was assigned to execution of the python code and for printing any screen output. Collaboratory also connects to the remote file service Google Drive. However, a drawback of Collaboratory is that use of the processor is rationed among users of the service, so computational time is constrained.

For nearly all analyses, the time-restriction for use of Colaboratory led to a restriction on analysis of the fruit fly hemibrain data to files less than 2.2 megabytes in size. These hemibrain data files often spanned both left and right hemispheres, but the majority of the records are assigned to the right hemisphere. Where the data file is about 2 megabytes, the analysis typically completed in a four-hour time frame, but a larger data file is expected to lead to an exceedingly long run time. The run time is further extended in the case of estimating values for the metric sigma, particularly where the network has a relatively large number of connections.

The files of the left hemisphere were generally smaller in size, and likewise, many of these data files contained an insufficient sample to estimate the metric values of interest. In this case, if the largest component of the network, by definition a contiguously connected set of neurons, had a count of neurons less than 36, then it is excluded from further evaluation. This criterion for neuron count per component was derived from practical testing of the method.

The excluded files of large size and without a hemisphere designation are FB and IB. These abbreviations are defined in Table 1. Likewise, the excluded files from the right brain hemisphere are aL, AL, AOTU, AVLP, bL, b’L, CA, CRE, gL, ICL, LAL, LH, LO, PED, PLP, PVLP, SCL, SIP, SLP, SMP, SPS, and WED; and from the left hemisphere, SMP was excluded.

Included files in the analysis, along with a designated hemisphere in parentheses, are: AB(L), AB(R), aL(L), AL(L), AME(R), ATL(L), ATL(R), a’L(L), a’L(R), bL(L), BU(L), BU(R), b’L(L), CAN(R), CRE(L), EB, EPA(L), EPA(R), FLA(R), gL(L), GNG, GOR(L), GOR(R), ICL(L), IPS(R), LAL(L), LOP(R), ME(R), NO, PB, SAD, SCL(L), SIP(L), SPS(L), VES(L), VES(R). This is a total of 36 regions of interest. This is a smaller portion of the hemibrain data as available to this study, and in addition, the hemibrain data project is limited to over 20,000 neurons, a subsample of the fruit fly brain. Overall, the fruit fly brain is a neural system spanning 250 μm and contains over 100,000 neurons [14].

Therefore, the main focus of this empirical analysis is to verify and refine the methodology for the network search procedure of square grid-ness, and secondarily to observe the prevalence of these square grids in the fruit fly data samples. This procedure and its code may be modified by others for identification of other kinds of network patterns of interest.

## 3. Results

### 3.1. Assessing Parameters of the Simulated Networks

The simulated graphs and their related metric values are shown in Table 2. The graphs tested were the completely connected network, the square grid lattice, the triangular grid lattice, the randomly connected network, the network based on a preferential attachment model, and the network based on a small-world model.

The grid-like networks were simulated with a range of 9 to 81 nodes, while the non-grid networks were simulated with a range of 9 to 27 nodes (Table 2). This procedure allows for observation of metric values across networks that range by size, thereby leading to more robust criteria for detection of a square grid-like pattern. The goal is to find as many square grid-like patterns in a network as possible, while minimizing the possibility of falsely identifying non-square grid patterns as square grids.

The completely connected network is modeled by a single parameter, node count, since the model stipulates that all nodes are interconnected. The square and triangular grid networks are also defined by node count. The randomly connected network has an additional parameter for probability of a new connection, and its values were varied at 0.4 and 0.5 (Table 2). If a lower value is used, outside this range, then it may generate a network that is not contiguous, so the network is formed as a set of unconnected components. These sets of values were also chosen for creating a sufficient density of connections, comparable to the corresponding connection density in a grid-like network.

The preferential attachment model also includes a parameter, M, for preferential attachment of new nodes to existing nodes in the network, and that is specifically defined in NetworkX [16], and the literature [15]. The networks for this model were simulated where the above parameter M varied from 3 to 6 (Table 2). Lastly, simulated networks were generated for a small-world architecture. This model has a parameter, k, corresponding to the number of nearest neighbors in a ring topology [16], and this was varied from 4 to 6 (Table 2). There is also a second parameter in the small-world case, labeled by P, for probability of rewiring an edge [16]. These networks were simulated with a *p* value of 0.1 and 0.5 (Table 2).

The metrics of interest include the square clustering coefficient [17], transitivity, bipartivity, and sigma (Table 3). The clustering coefficient values do not provide a guide for identification of the above models of simulated networks. However, transitivity has values of interest. The square grid lattices show a transitivity of 0.0, while the others show fairly high values on average, typically around an average value of 0.5. Likewise, the triangular grid networks generally clustered with values that ranged from 0.42 to 0.49.

Bipartivity is useful for identification of a square grid network pattern, and in fact, the generated square grid networks showed a bipartivity value of 1.0. The other networks also showed a potential for high values of bipartivity, many with a value near 1.0, but this is expected since a model may generate patterns, such as the square grid-like, by chance.

Next, the sigma parameter measured the fit of the network to a small-world model. Values of sigma that are larger than 1.0 [18] are evidence of a small-world network architecture. As expected, the generated small-world networks are associated with high values of sigma, some cases with a value near 5.0. Likewise, the triangular grid lattices show high values of sigma, not unexpected since this parameter relies on quantifying the occurrence of triangular grids in a graph, so a network of triangular grids may fit with the definition of a small-world architecture.

Transitivity is another metric employed for the square grid search. The criterion, in this case, ranged from a value of 0.0 to 0.20 (Table 3). The value of 0.0 is consistent with a perfectly formed square grid arrangement, while values greater than 0.20 would detect triangular grids for exclusion. Next, the criterion for bipartivity ranged in value from 0.80 to 1.0 (Table 3), and serves as another filter in this procedure. There are network types, such as random and small-world, that are not filtered by this criterion, but altogether, these metrics provide values that increase the precision in the search for square grid-ness. Lastly, the criterion for sigma was restricted to values between 0.0 and 0.50 (Table 3). These values filter to separate square grids from non-square grid arrangements as supported by the simulation results (Table 2).

### 3.2. Square Grid Arrangement in the Fruit Fly Brain

The search of the fruit fly hemibrain resulted in 46 of the windows meeting the criteria for square grid-ness (Supplementary Materials). Overall, the windows in general represent all possible subnetworks of a network by region of interest (ROI), where each subnetwork includes a neuron, along with all neighboring neurons within a span of two hops. The procedure is further described in the methods. The following ROIs correspond to the above 46 windows with the attribute of putative square grid-ness: FLA(R), GNG, GOR(L), GOR(R), ICL(L), ME(R), PB, SAD, SCL(L), and SIP(L).

**Figure 4:**
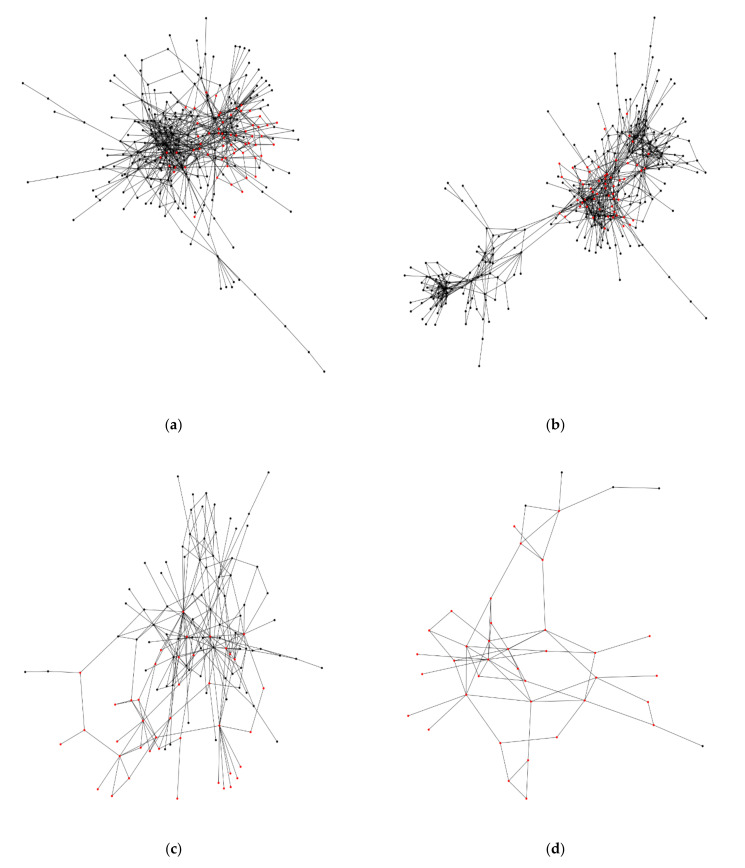

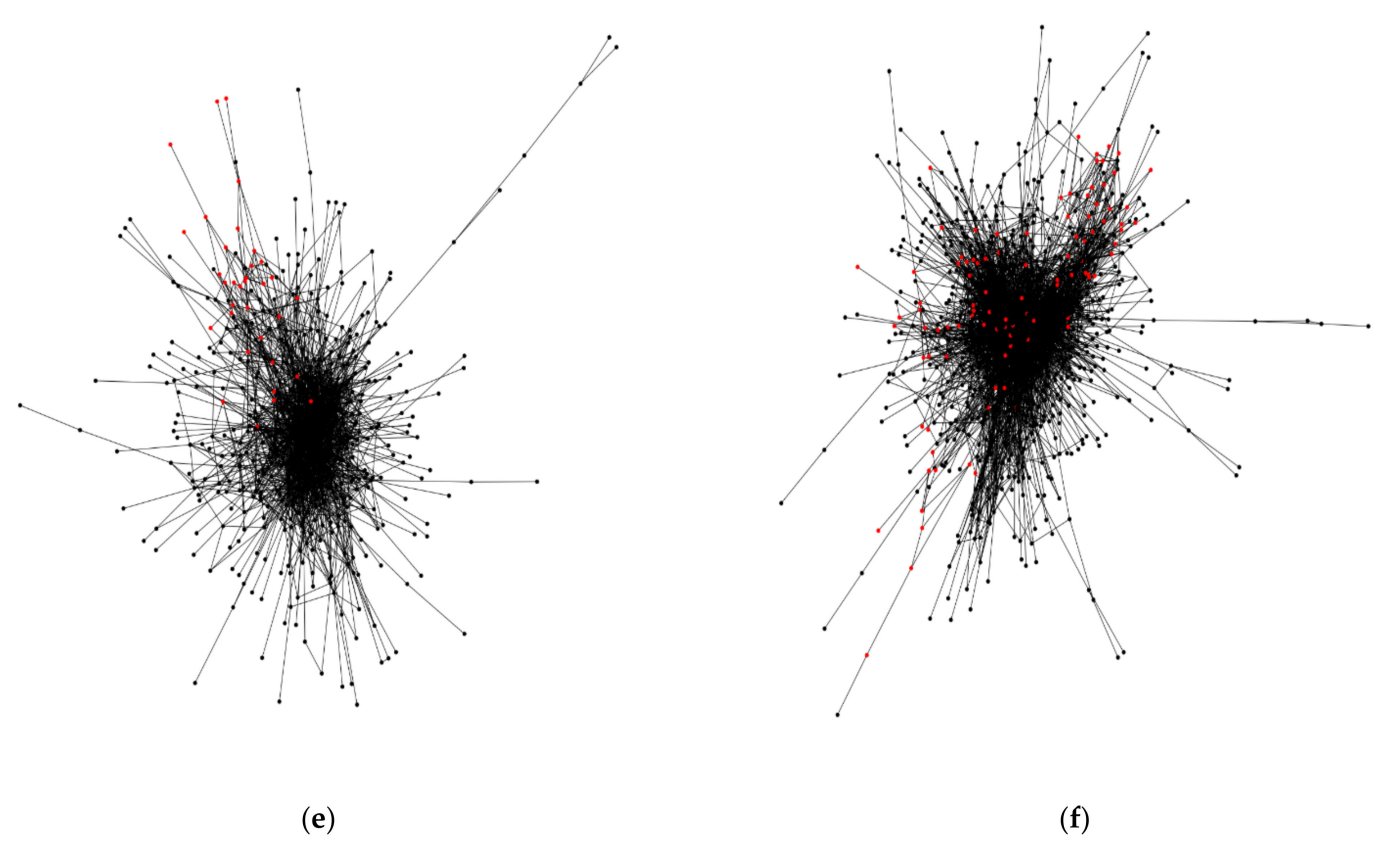
The major network component of each fruit fly brain region that contains more than one window with a putative square grid arrangement. The red color nodes indicate membership of neurons in a putative square grid arrangement, based on the data of the 46 subnetworks (Supplementary Materials; Section 3.2). The nodes in black color are neurons that are not members of a putative square grid. The edges are the synaptic connections. (**a**) Cantle region in the right hemisphere; (**b**) epaulette region in the left hemisphere; (**c**) flange region in the right hemisphere; (**d**) medulla region in the right hemisphere; (**e**) superior clamp region in the left hemisphere; (**f**) superior intermediate protocerebrum in the left hemisphere.

The following eight ROIs include a single window of square grid-ness: ATL(R), EPA(R), GNG, GOR(L), GOR(R), ICL(L), PB, and SAD. These windows typically include one or only a few examples of single square grids, and likewise, the count of neurons is also typically low in number (< 20). Six other ROIs have more than one window with a putative square grid arrangement: CAN(R), EPA(L), FLA(R), ME(R), SCL(L), and SIP(L) (Figure 4). For CAN(R), there are 60 nodes of square grid-ness among a total of 278 nodes (square grid-ness score = 22%). These nodes are derived from the windows which also show the attribute of overlapping. Likewise, the others have these ratios of square grid-ness by ROI: EPA(L) 55/318 = 17%; FLA(R) 41/124, ME(R) 36/41, SCL(L) 34/534, and SIP(L) 98/898.

If the windows of putative square grid-ness are not contiguous in the network, then they are not considered evidence of a square grid arrangement of interest, but instead alternate explanations exist, such as a random occurrence in generation of the network, or bias in sampling of a small network.

The fruit fly hemibrain project [14] is mainly derived from the central dense portion of the right brain hemisphere. Figure 4a shows the neural networks of the cantle region in the right hemisphere, and highlights the nodes, representing the neurons, that participate in a square grid arrangement (22%; see above). A visual inspection of these nodes shows that they appear to show a larger scale clustering of square grid-ness. Figure 4b of the epaulette (EPA) region in the left hemisphere shows a similar result (17%). Even though this region is in the left hemisphere, the data sample of neurons is larger than the cantle (CAN) region in the right hemisphere. Overall, these results are specific to the major component of each region’s neuronal network, so that the connections are contiguous as a whole, an assumption inherent to many of the network metrics.

Figure 4c,d show a smaller sample of neurons in the flange (FLA) and medulla (ME) regions, respectively. The flange region has a major component of 124 nodes, yet has a high proportion of square grid-ness (33%). Likewise, the medulla region has a small number of nodes, 41, and a very high proportion of square grid-ness (88%). This could result from sampling bias, but an expectation of this bias would require a higher frequency of square grids across the brain regions. Lastly, Figure 4e,f show regions in the left hemisphere with a large number of neurons: the superior clamp (SCL) region with 534 nodes, and the superior intermediate protocerebrum (SIP) region with 898 nodes (square grid-ness at 6% and 11%, respectively).

## 4. Discussion

Given the criteria for finding square grid arrangements in the fruit fly hemibrain data set [14], six of the regions show square grids that cluster in the neural network (Figure 4). These kinds of grid arrangements are likewise observed in empirical and simulation work [6,11,22]. The application of this search method to the fruit fly brain is not thorough because the hemibrain data set [14] does not fully cover the neuronal network of the brain, and furthermore, the larger files were excluded because of incomputability. However, the six neural network regions (Figure 4) provide qualitative support for the hypothesis of square grid-ness at the expected scale of size. This observation contrasts with a case where the square grid members do not cluster in the network, an occurrence which is not readily supported (Figure 4).

Additionally, of the 36 analyzed regions of the fruit fly brain, a majority of these regions did not contain a square grid arrangement or did not meet the criteria for square grid-ness, such as resulting from a small data sample.

To further provide evidence that square grids are not from a random expectation, there was a search of functionality for the above six fruit fly brain regions (Figure 4). However, the functions of these regions are mostly unknown [23], and moreover, the local scale of the square grids would require an even finer scale of understanding of the animal neural system.

For the case of the mushroom body, there is evidence of involvement in higher order processing of sensory information [24,25,26], but no square grid-ness was detected in the sample used from that larger region. Another large-scale structure involved in higher order information processing is the central complex [27]. This also had no square grid-ness as exemplified by the noduli region. Lastly, the medulla region showed square grid-ness (Figure 4d), a member of the optic lobe, but the sample size is insufficient to interpret with any confidence. The optic lobe is involved in visual information processing, a function that involves 60% of the neurons in the fruit fly brain [28]. For further reference, the anatomical scheme for these regions is described by Ito and others [19].

While the network structure is expected to influence the flow of information from an abstract perspective [11], this influence is also observed in the neural systems of animals. For example, as in many other animals, the fruit fly navigates its external world by visual information processing, and therefore, its neural system is expected to segment a scene into parts and store the internal representations of a scene into memory. Animals, such as the fruit fly, are expected to converge on the same or similar solutions for visual input, and likewise, reflect commonality in the underlying neuronal network. This expectation is seen in the convergence between two unrelated animal clades in the sharing of neural structure of an early pathway of vision [29], even though their eyes are formed by a different design (compound versus camera eye).

In conclusion, there is support for clustering of square grids in the sampled regions of the fruit fly brain. If instead these square grids are from a random occurrence, then they are expected to scatter into a non-clustered-like arrangement. This study also offers a procedure with code to search for patterns in other neural networks. In addition, it is probable that grid arrangements are markers for division in task for the processing of information [6,11], and this study’s approach is one method to observe the neural network architecture and hypothesize on functions across the parts of a neural system.

## Figures and Tables

**Table 1:** Nomenclature for fruit fly hemibrain by synapse-rich neuropil, brain region and its abbreviation [19]. The list is restricted to regions which are referenced in this paper. Abb. = abbreviation of brain region name.

Neuropil	Brain Region	Abb.	Neuropil	Brain Region	Abb.
Optic Lobe	Medulla	ME	Lateral Horn	Lateral Horn	LH
	Accessory Medulla	AME	Superior Neuropils	Superior Lateral Protocerebrum	SLP
	Lobula	LO		Superior Intermediate Protocerebrum	SIP
	Lobula Plate	LOP		Superior Medial Protocerebrum	SMP
Mushroom Body	Calyx	CA	Inferior Neuropils	Crepine	CRE
	Pedunculus	PED		Superior Clamp	SCL
	Alpha Lobe	aL		Inferior Clamp	ICL
	Alpha Prime Lobe	a’L		Inferior Bridge	IB
	Beta Lobe	bL		Antler	ATL
	Beta Prime Lobe	b’L	Antennal Lobe	Antennal Lobe	AL/AL2
	Gamma Lobe	gL	Ventromedial Neuropils	Ves	VES
Central Complex	Fan Shaped Body	FB		Epaulette	EPA
	Asymmetric Body	AB		Gorget	GOR
	Epsilloid Body	EB		Superior Posterior Slope	SPS
	Protocerebral Bridge	PB		Inferior Posterior Slope	IPS
	Noduli	NO	Periesophageal Neuropils	Saddle	SAD
Lateral Complex	Bulb	BU		Flange	FLA
	Lateral Accessory Lobe	LAL		Cantle	CAN
Ventrolateral Neuropils	Anterior Optic Tubercle	AOTU	Gnathal Ganglia	Gnathal Ganglia	GNG
	Anterior Ventrolateral Protocerebrum	AVLP			
	Posterior Ventrolateral Protocerebrum	PVLP			
	Posteriorlateral Protocerebrum	PLP			
	Wedge	WED			

**Table 2:** Parameter values of simulated networks.

Network Model	Node Count	Model Parameters	Clustering Coefficient (Min, Max)	Transitivity (Min, Max)	Bipartivity (Min, Max)	Sigma (Min, Max)
Complete	9 to 27	-	1.0, 1.0	1.0, 1.0	0.5, 0.5	1.0, 1.0
Square grid	9 to 81	-	0.16, 0.28	0.0, 0.0	1.0, 1.0	0.0, 0.0
Triangular grid	9 to 81	-	0.13, 2.0	0.42, 0.49	0.63, 0.70	1.26, 3.96
Random	9 to 27	P = 0.4, 0.5	0.07, 0.40	0.14, 0.60	0.50, 0.93	0.89, 1.23
Preferential attach	9 to 27	M = 3 to 6	0.15, 0.56	0.20, 0.67	0.50, 0.65	0.75, 1.08
Small world	9 to 27	k = 4 to 6 P = 0.1, 0.5	0.05, 0.55	0.08, 0.72	0.54, 0.89	0.60, 4.88

**Table 3:** Parameter values for an idealized square grid network arrangement.

Parameter	Utility of Parameter	Parameter Values
Node Count		36 to ∞
Transitivity	Filter for triangular grid	0.0 to 0.20
Bipartivity	Filter for square grid	0.80 to 1.0
Sigma	Filter for non-square grid	0.0 to 0.50

## Data Availability

All data reported are contained in the listed references and in the Supplementary Materials.

## Supplementary Materials

Python code and images of square grid results are available online at https://www.mdpi.com/article/10.3390/neurosci3010007/s1.
