# Supplementary figures and images for: Detecting Square Grid Structure in an Animal Neuronal Network
Source: NeuroSci. 2022 Feb 3;3(1):91–103. doi: 10.3390/neurosci3010007 (PMC11523746; doi:10.3390/neurosci3010007)

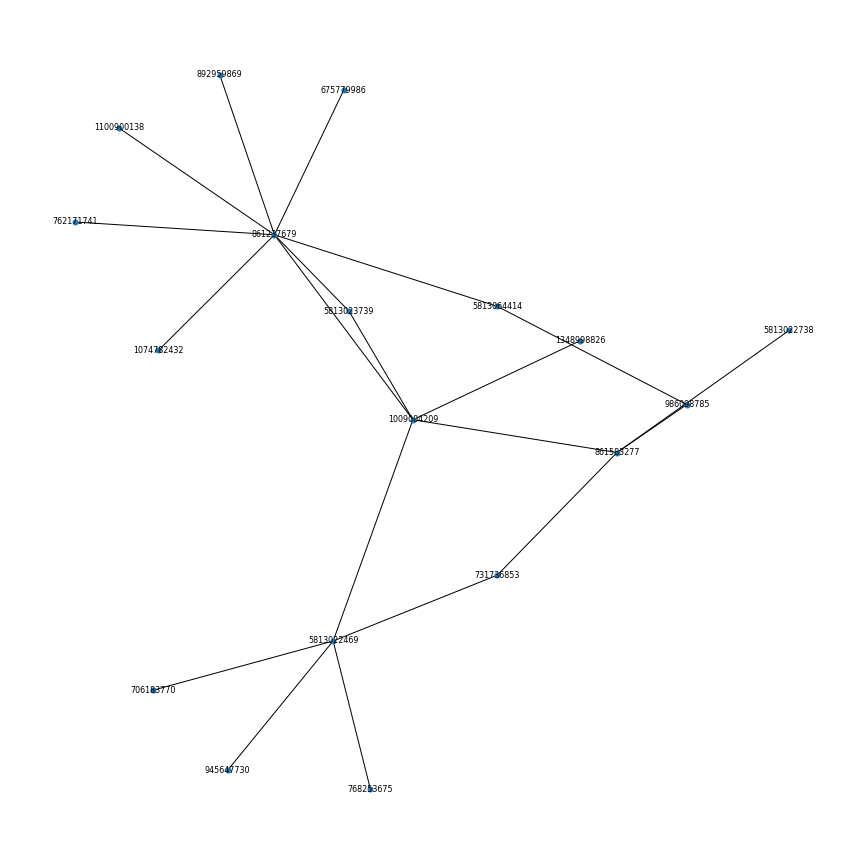

Supplement: Supplementary file 1 [file neurosci-03-00007-s001.zip › square_grid_windows/graph-ATL(R)-0-1009004209.png]

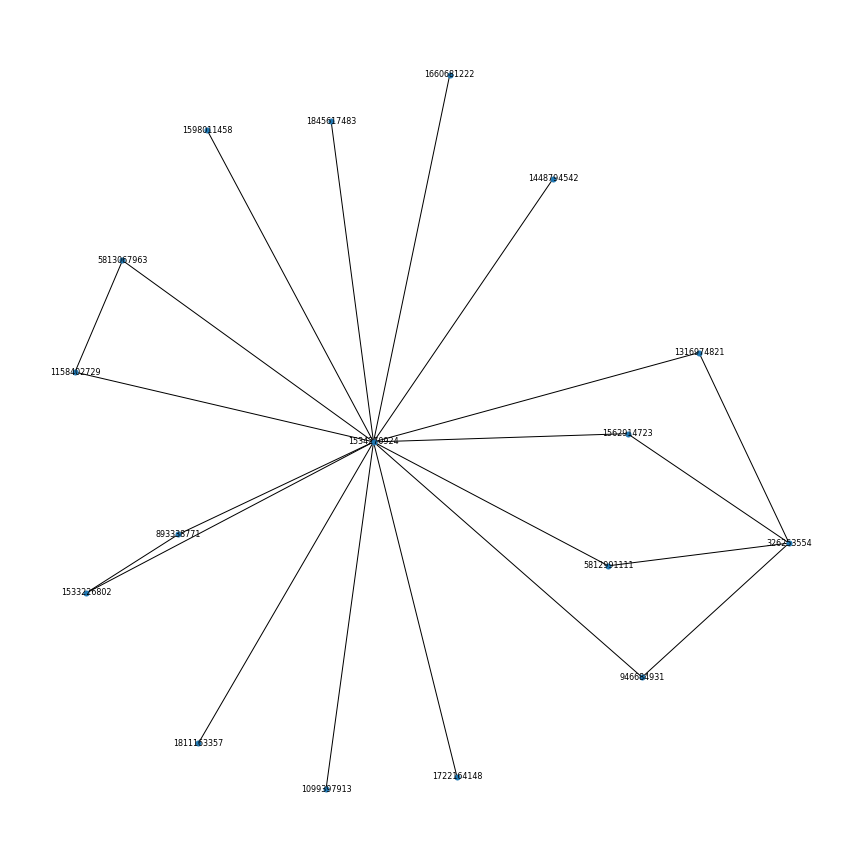

Supplement: Supplementary file 1 [file neurosci-03-00007-s001.zip › square_grid_windows/graph-CAN(R)-0-1316974821.png]

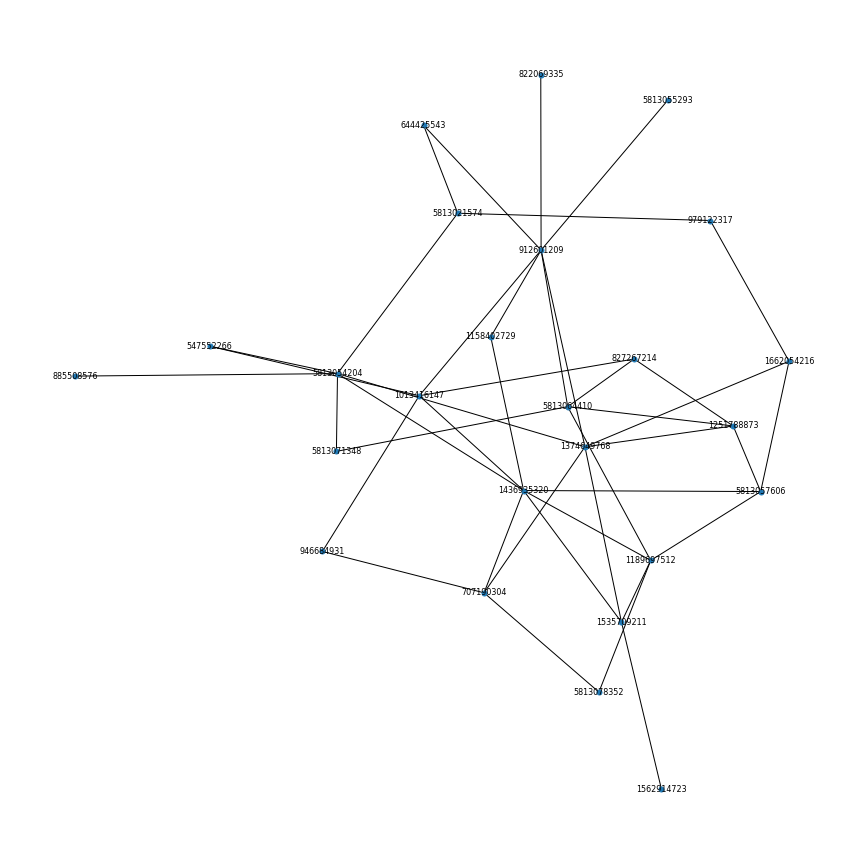

Supplement: Supplementary file 1 [file neurosci-03-00007-s001.zip › square_grid_windows/graph-CAN(R)-0-1374649768.png]

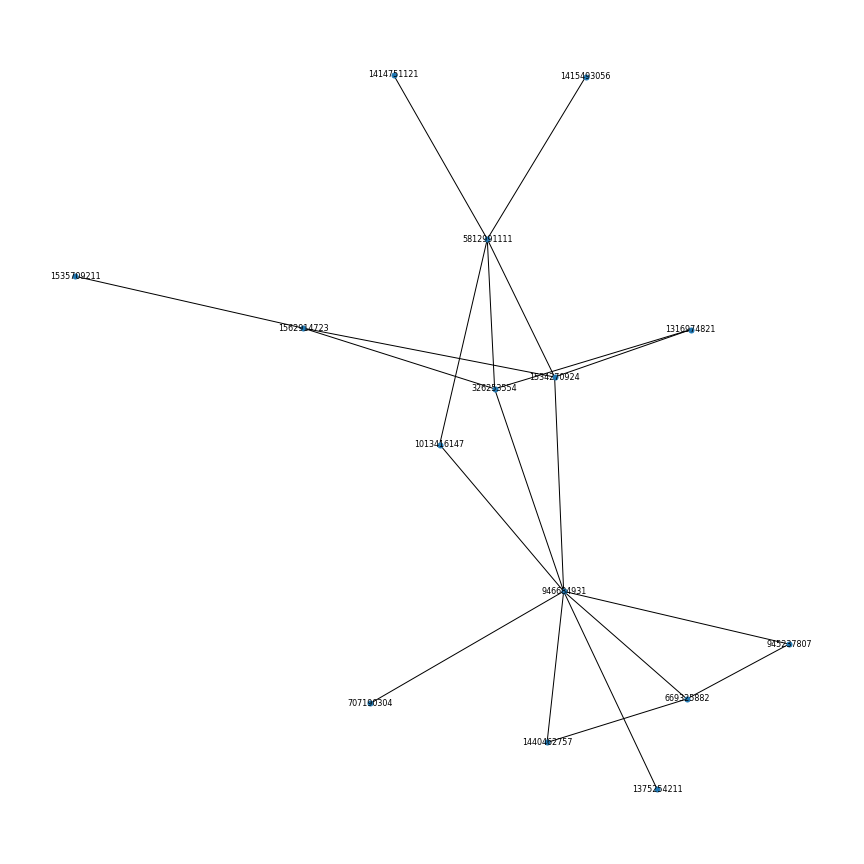

Supplement: Supplementary file 1 [file neurosci-03-00007-s001.zip › square_grid_windows/graph-CAN(R)-0-326253554.png]

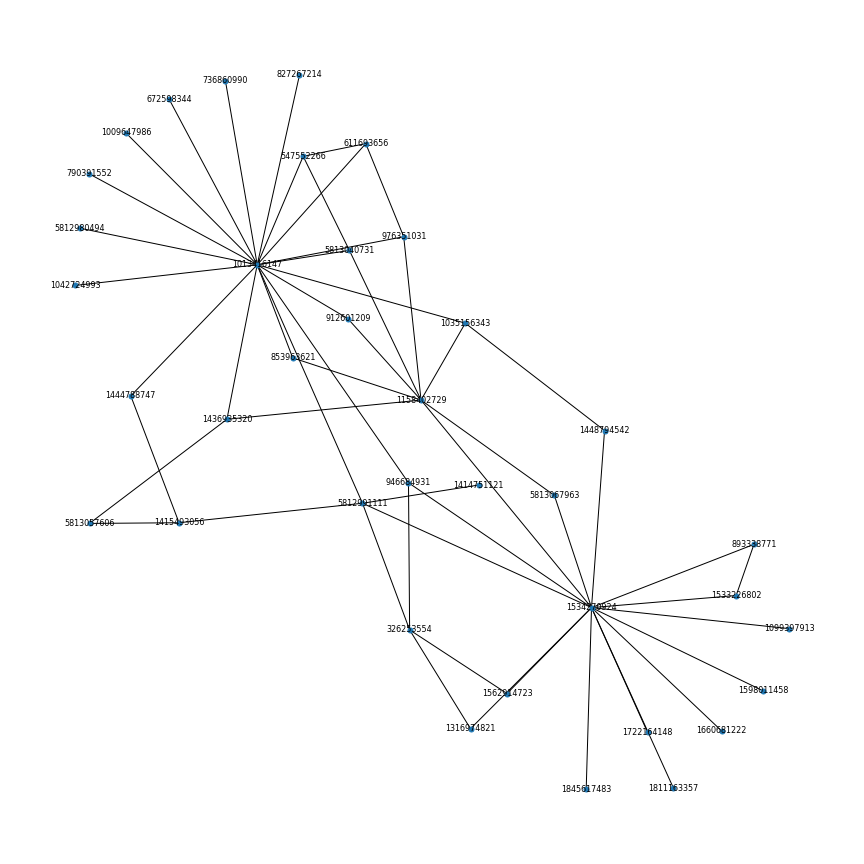

Supplement: Supplementary file 1 [file neurosci-03-00007-s001.zip › square_grid_windows/graph-CAN(R)-0-5812991111.png]

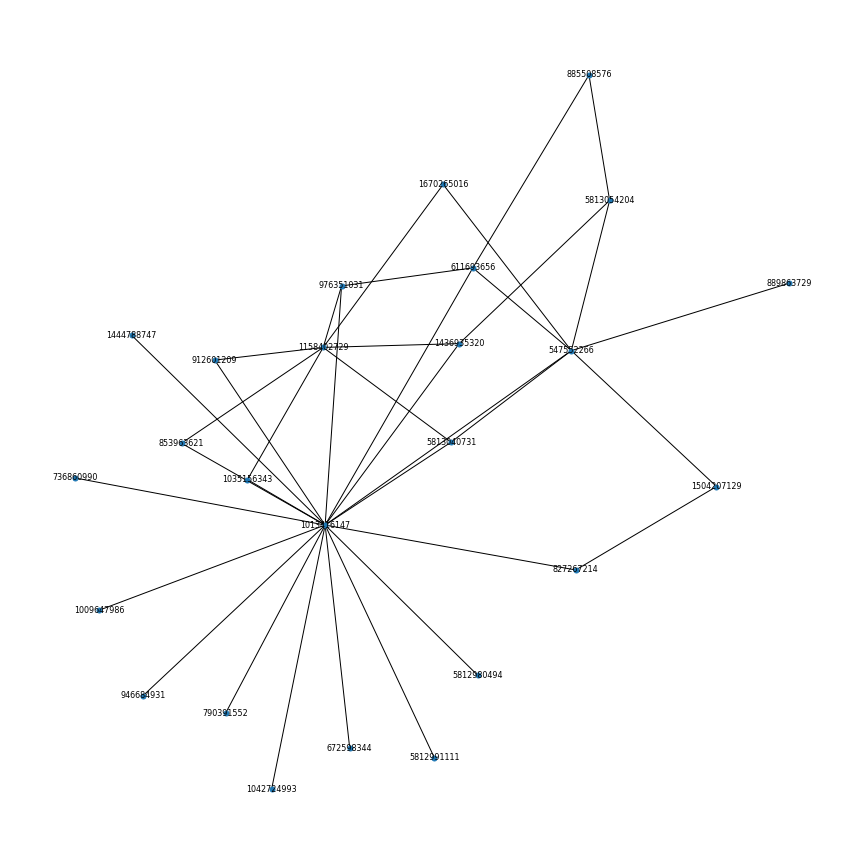

Supplement: Supplementary file 1 [file neurosci-03-00007-s001.zip › square_grid_windows/graph-CAN(R)-0-611693656.png]

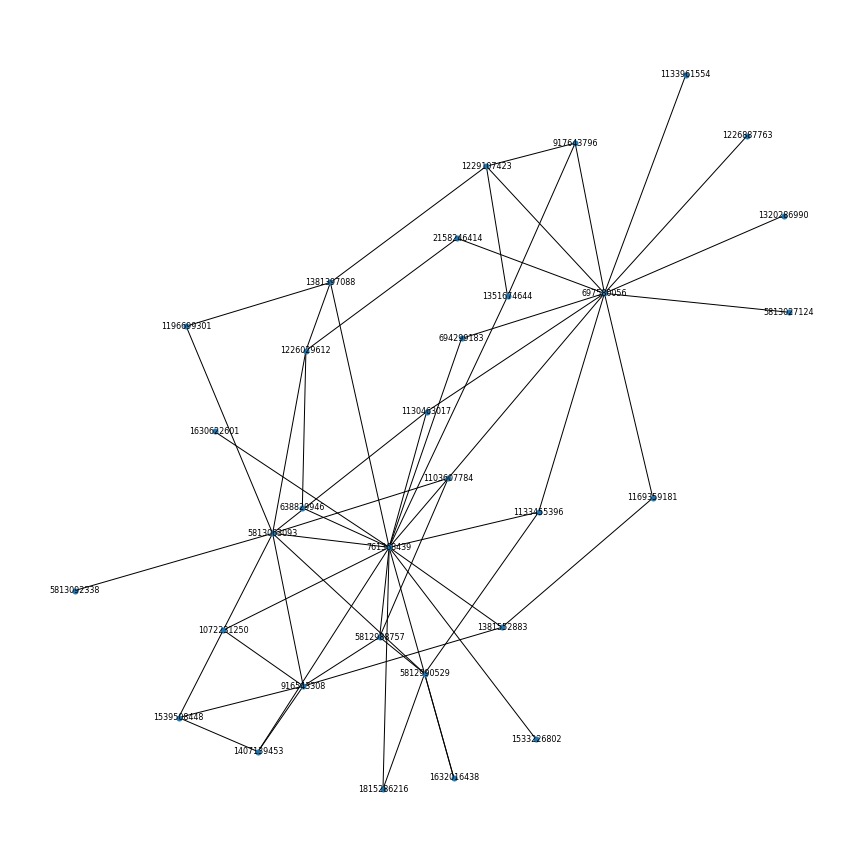

Supplement: Supplementary file 1 [file neurosci-03-00007-s001.zip › square_grid_windows/graph-EPA(L)-0-1130463017.png]

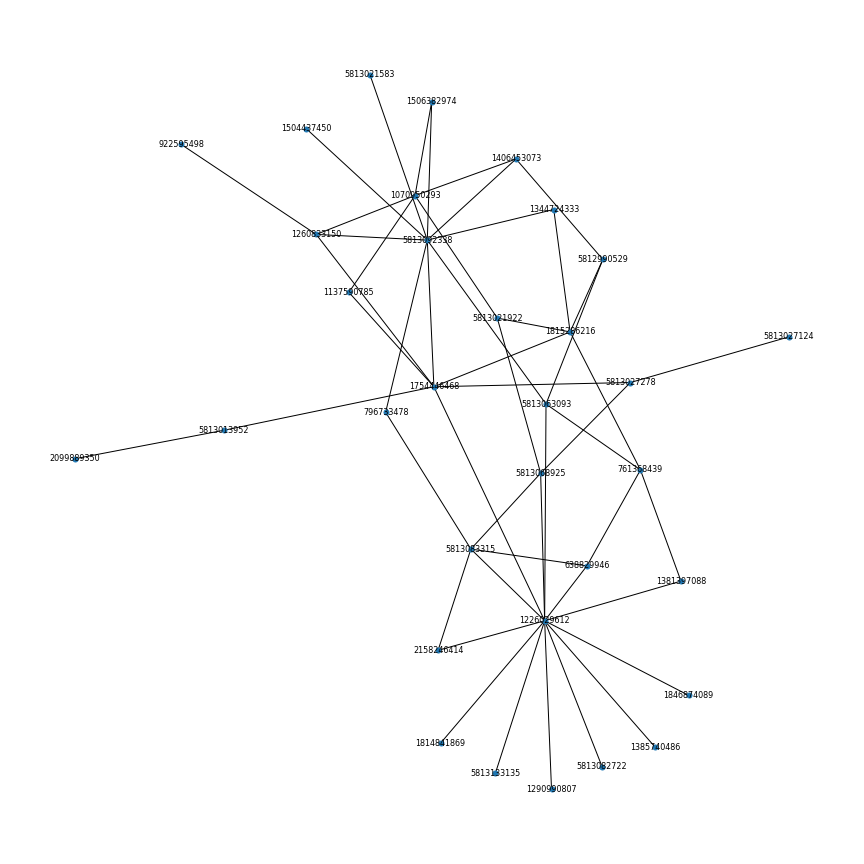

Supplement: Supplementary file 1 [file neurosci-03-00007-s001.zip › square_grid_windows/graph-EPA(L)-0-1754446468.png]

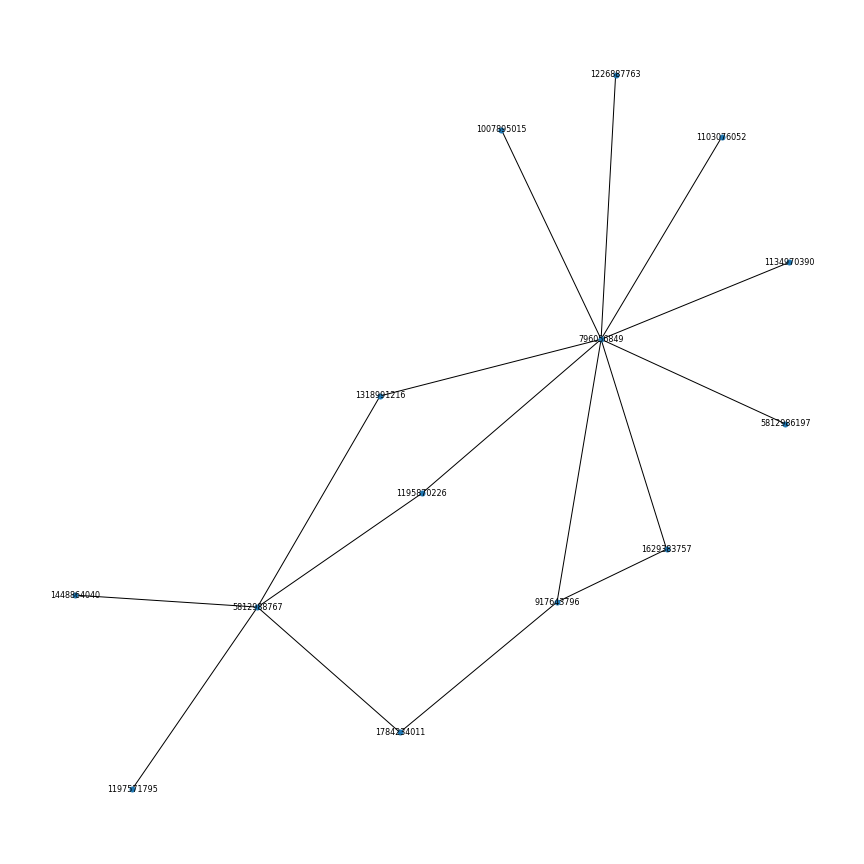

Supplement: Supplementary file 1 [file neurosci-03-00007-s001.zip › square_grid_windows/graph-EPA(R)-0-1195870226.png]

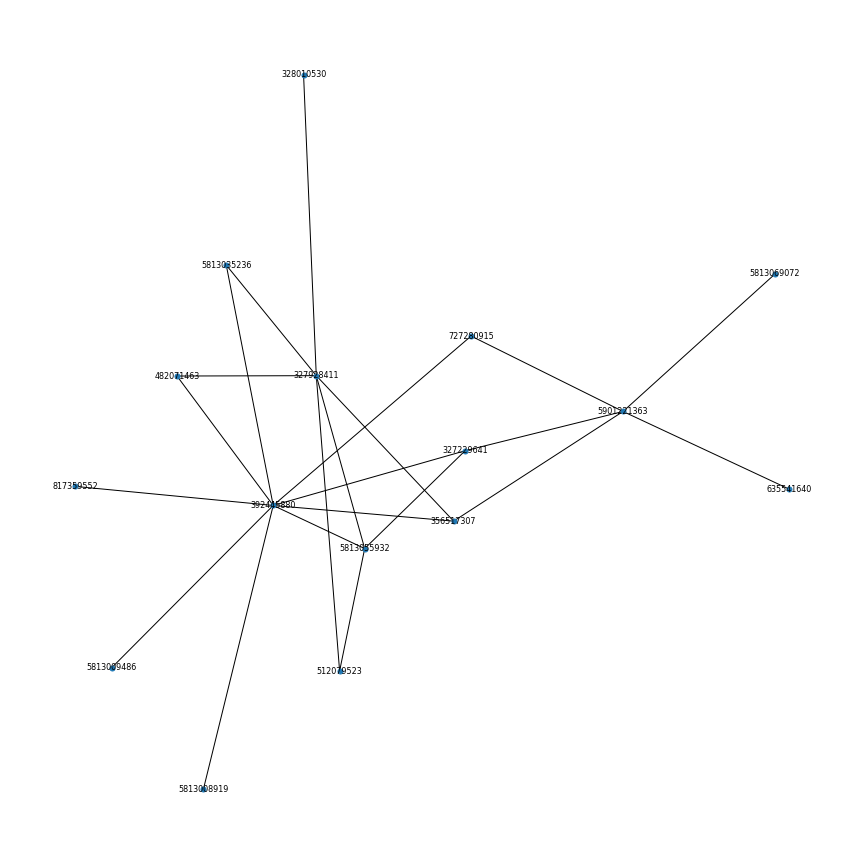

Supplement: Supplementary file 1 [file neurosci-03-00007-s001.zip › square_grid_windows/graph-FLA(R)-0-356517307.png]

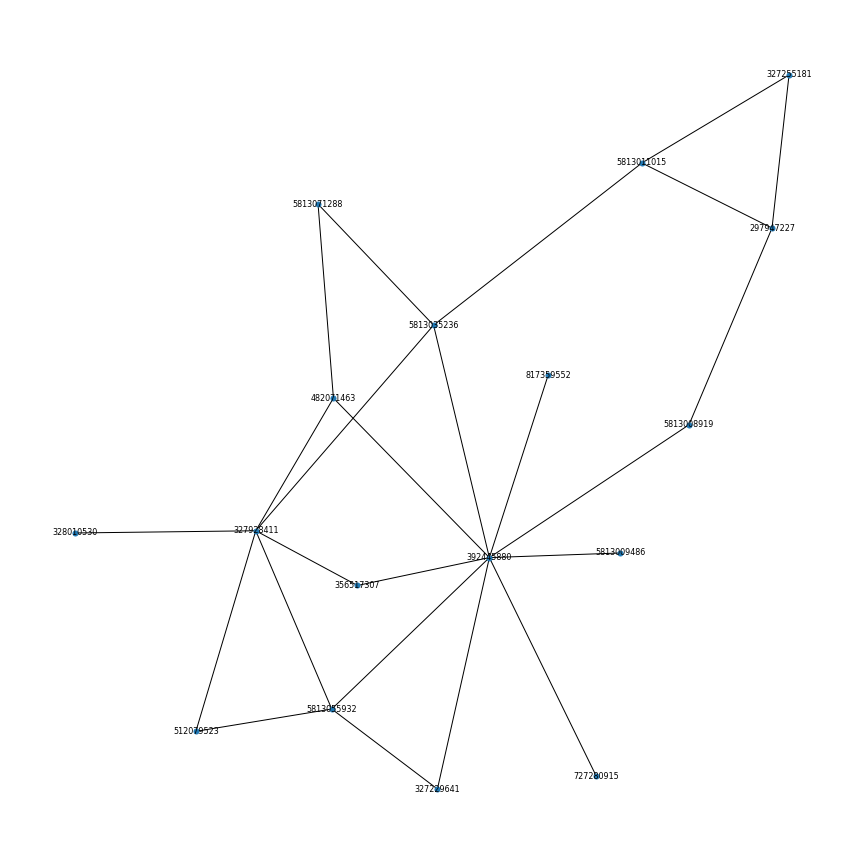

Supplement: Supplementary file 1 [file neurosci-03-00007-s001.zip › square_grid_windows/graph-FLA(R)-0-5813035236.png]

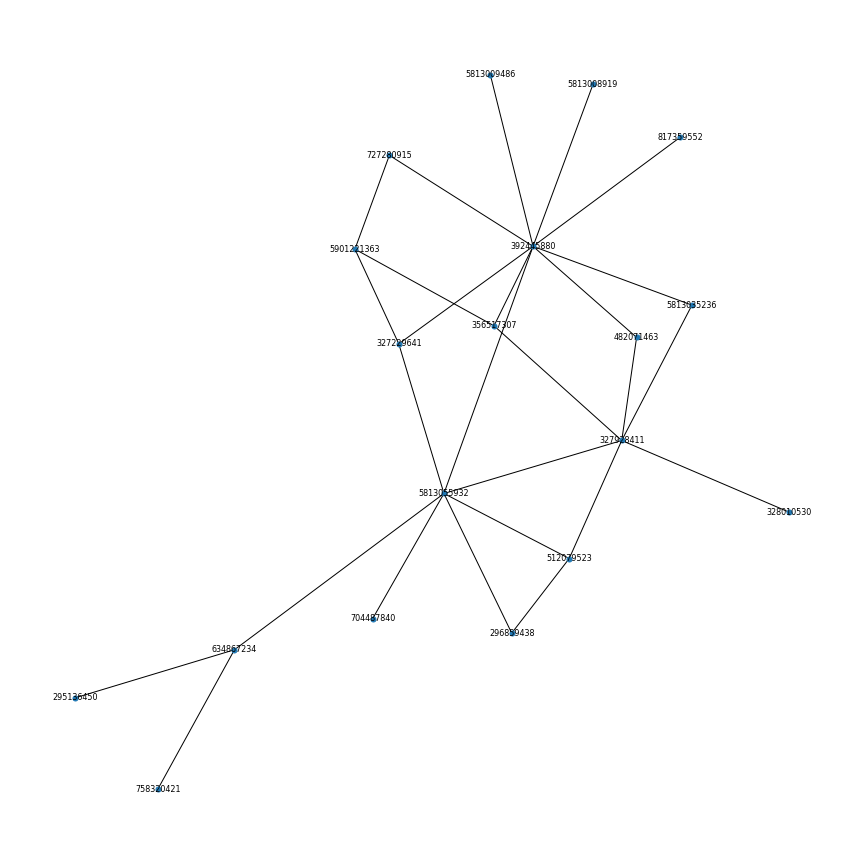

Supplement: Supplementary file 1 [file neurosci-03-00007-s001.zip › square_grid_windows/graph-FLA(R)-0-5813055932.png]

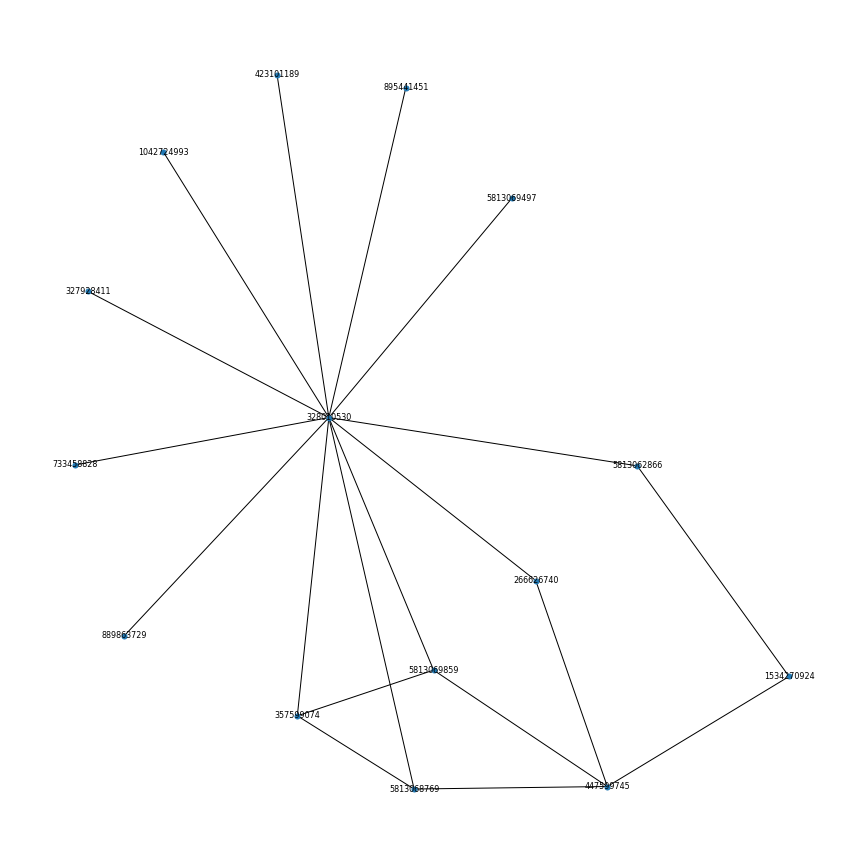

Supplement: Supplementary file 1 [file neurosci-03-00007-s001.zip › square_grid_windows/graph-FLA(R)-0-5813062866.png]

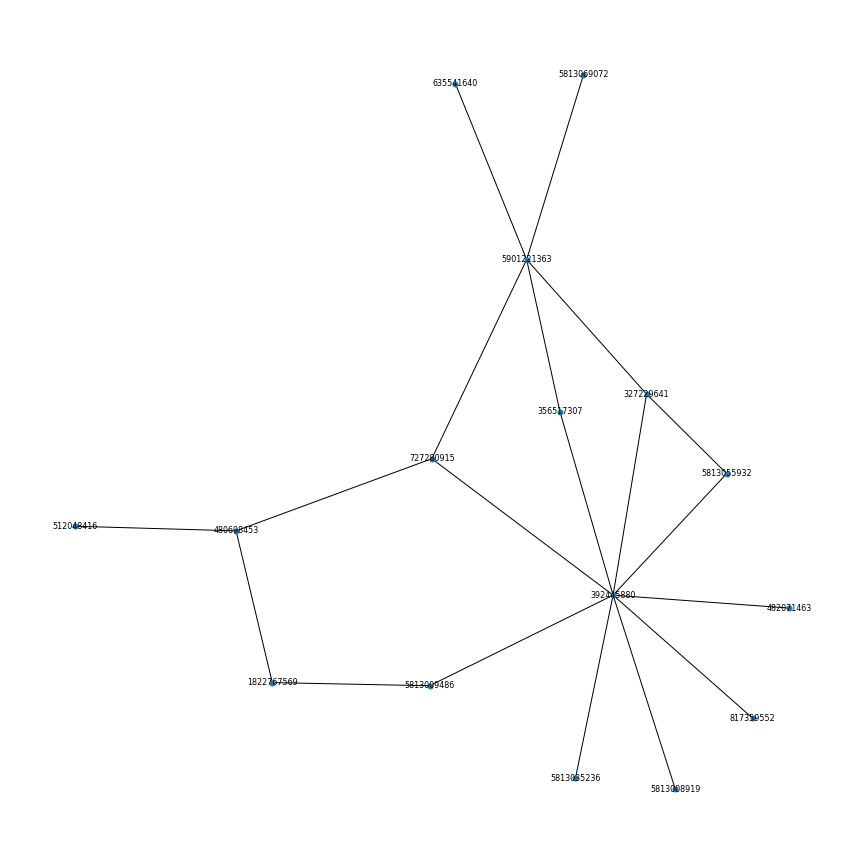

Supplement: Supplementary file 1 [file neurosci-03-00007-s001.zip › square_grid_windows/graph-FLA(R)-0-727280915.png]

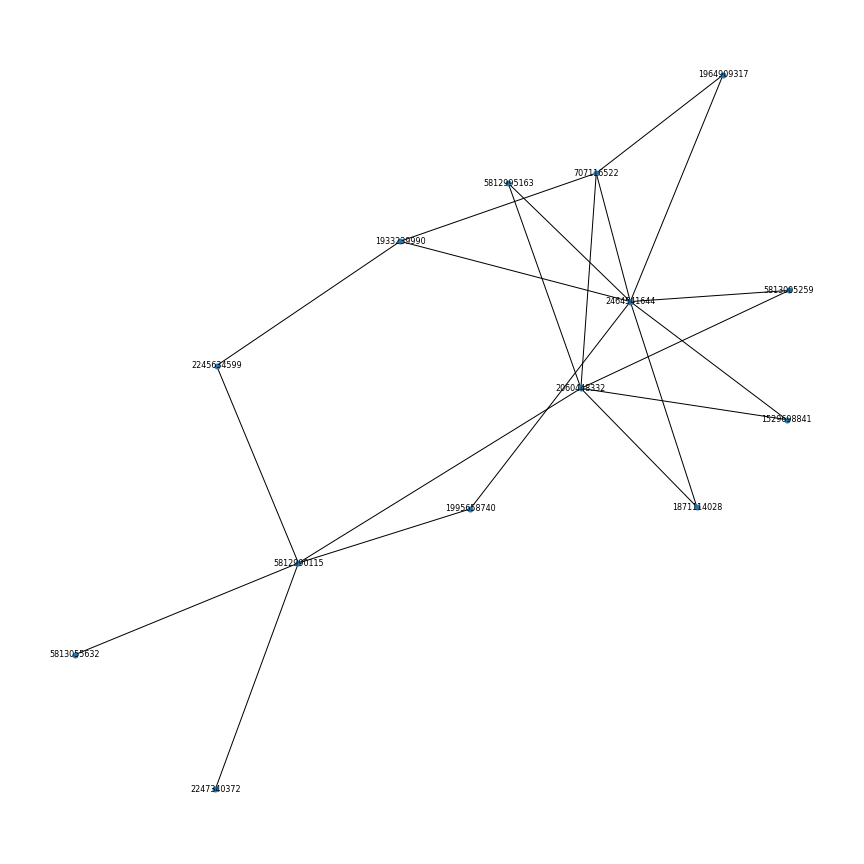

Supplement: Supplementary file 1 [file neurosci-03-00007-s001.zip › square_grid_windows/graph-GNG-0-1995658740.png]

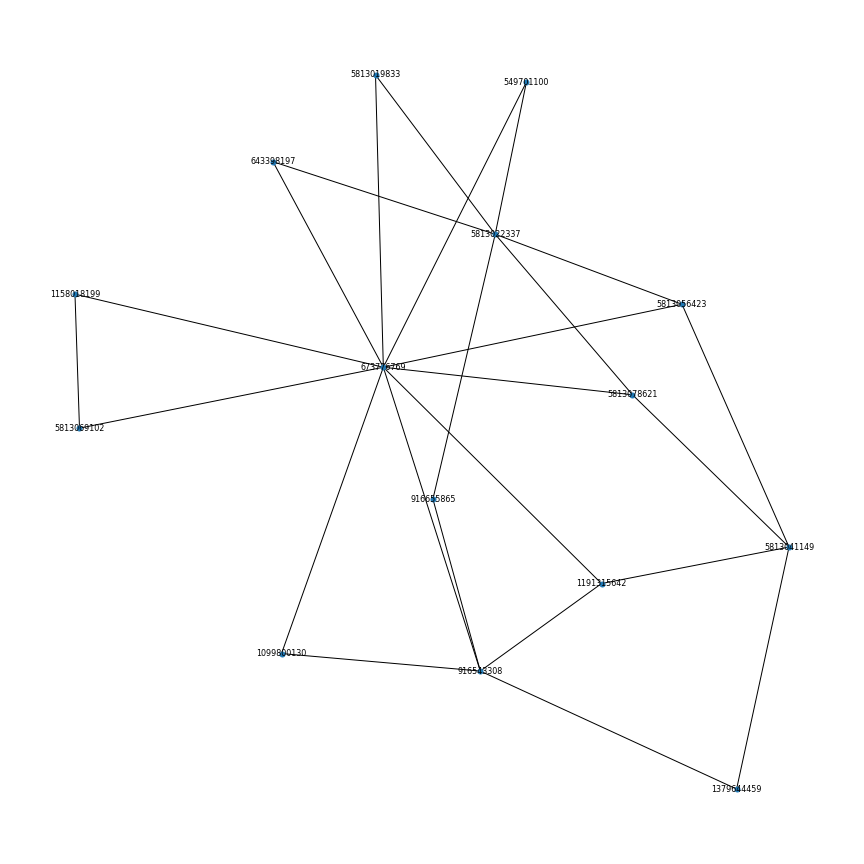

Supplement: Supplementary file 1 [file neurosci-03-00007-s001.zip › square_grid_windows/graph-GOR(L)-0-5813078621.png]

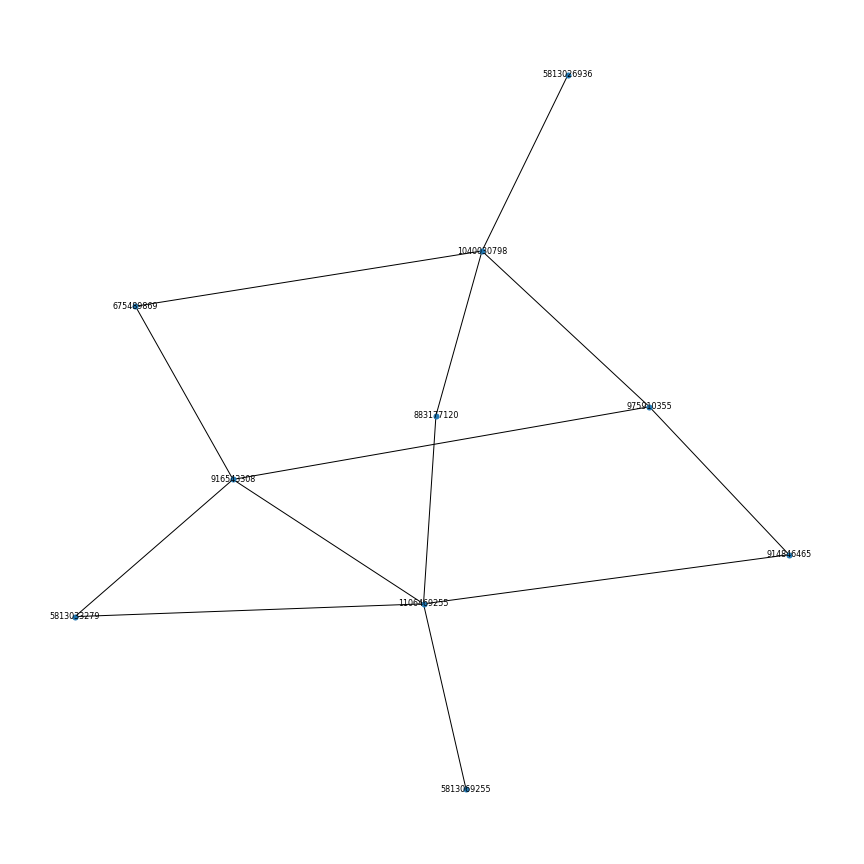

Supplement: Supplementary file 1 [file neurosci-03-00007-s001.zip › square_grid_windows/graph-GOR(R)-0-883177120.png]

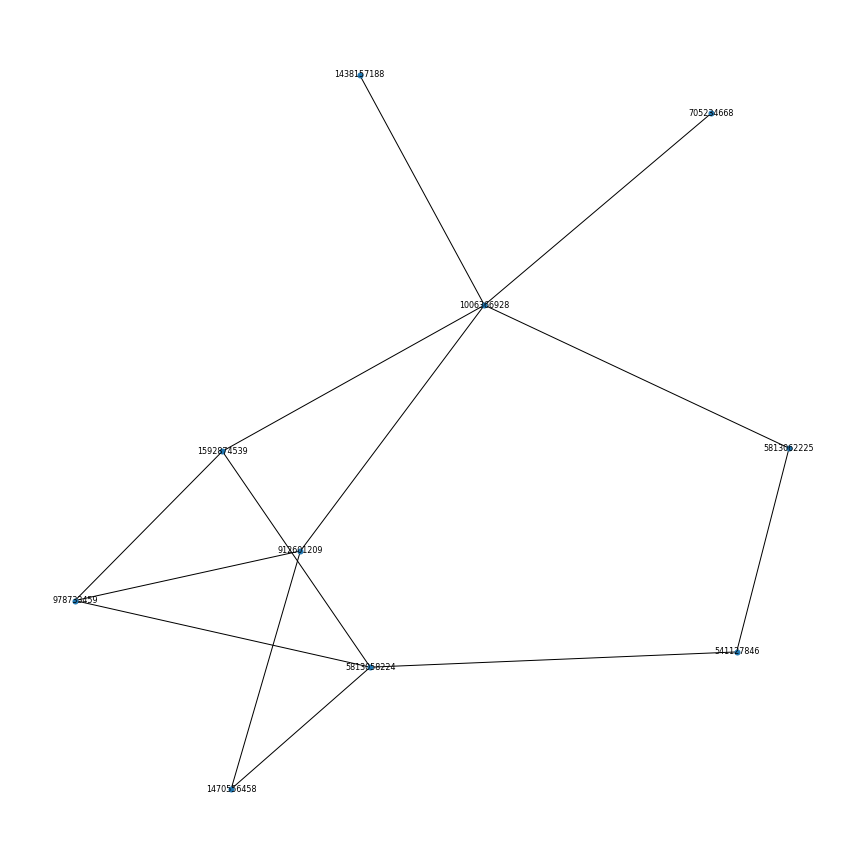

Supplement: Supplementary file 1 [file neurosci-03-00007-s001.zip › square_grid_windows/graph-ICL(L)-0-1592874539.png]

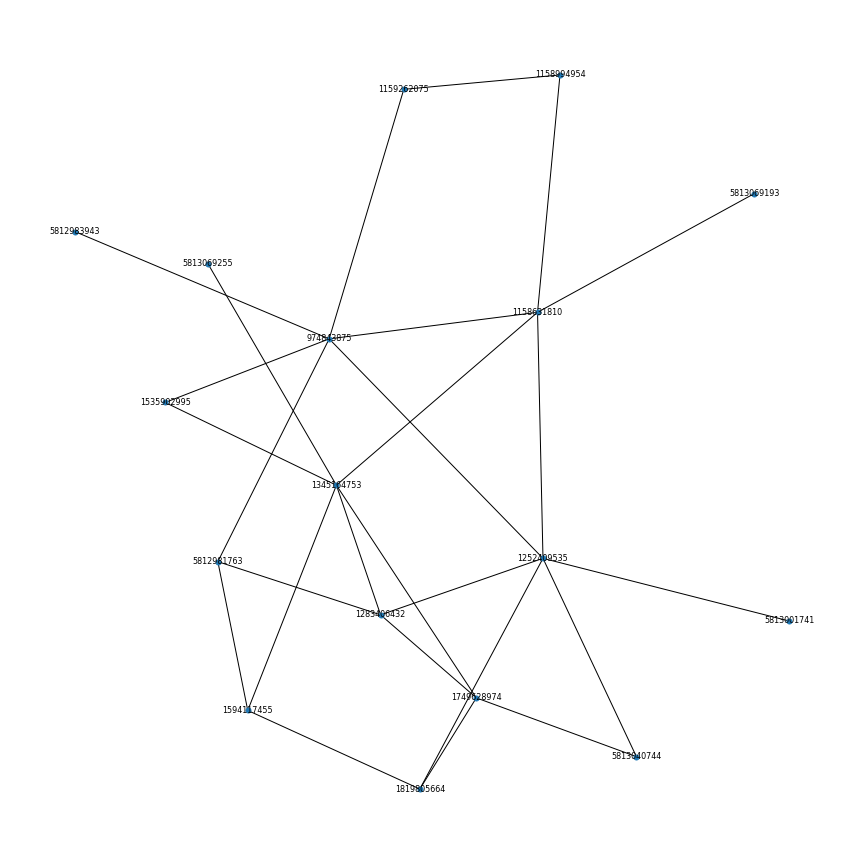

Supplement: Supplementary file 1 [file neurosci-03-00007-s001.zip › square_grid_windows/graph-ME(R)-1-1158631810.png]

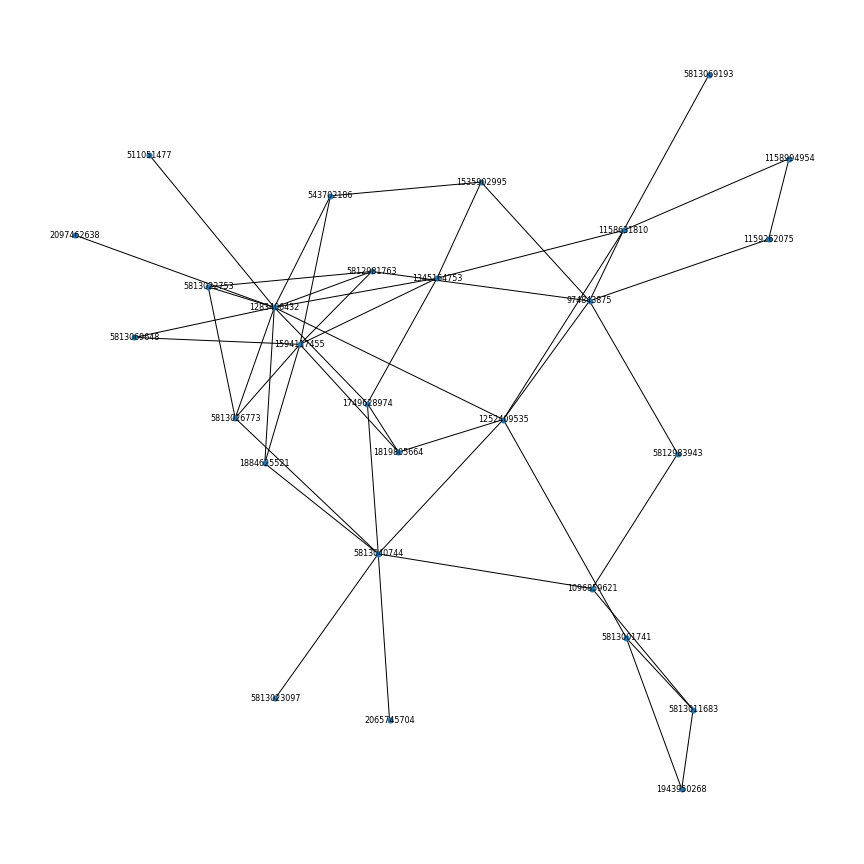

Supplement: Supplementary file 1 [file neurosci-03-00007-s001.zip › square_grid_windows/graph-ME(R)-1-1252409535.png]

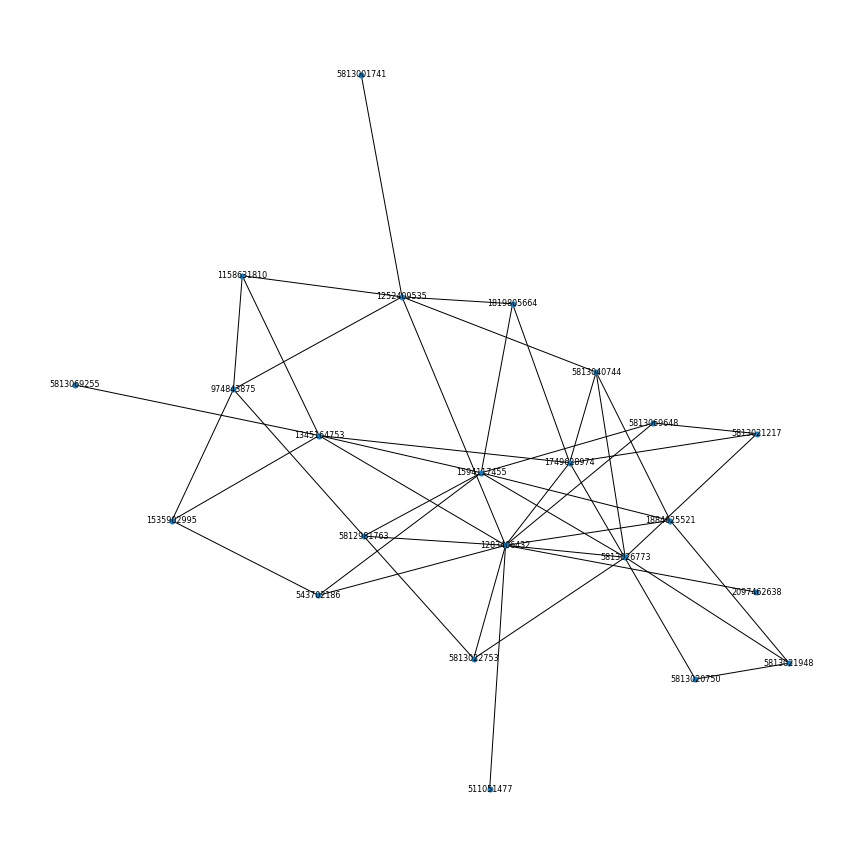

Supplement: Supplementary file 1 [file neurosci-03-00007-s001.zip › square_grid_windows/graph-ME(R)-1-1283406432.png]

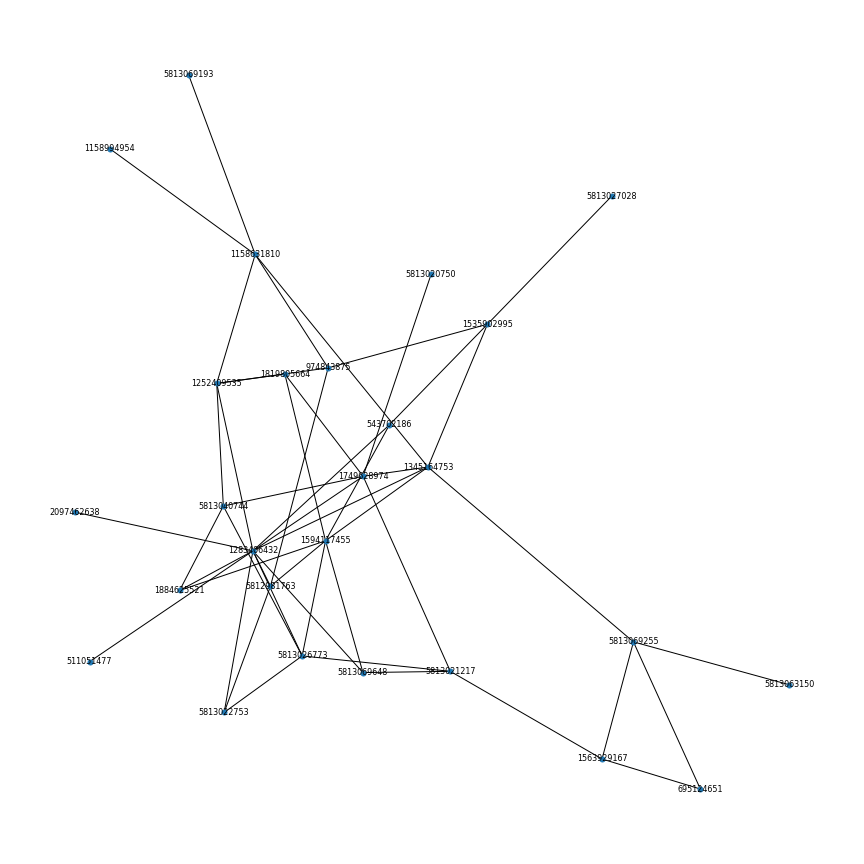

Supplement: Supplementary file 1 [file neurosci-03-00007-s001.zip › square_grid_windows/graph-ME(R)-1-1345164753.png]

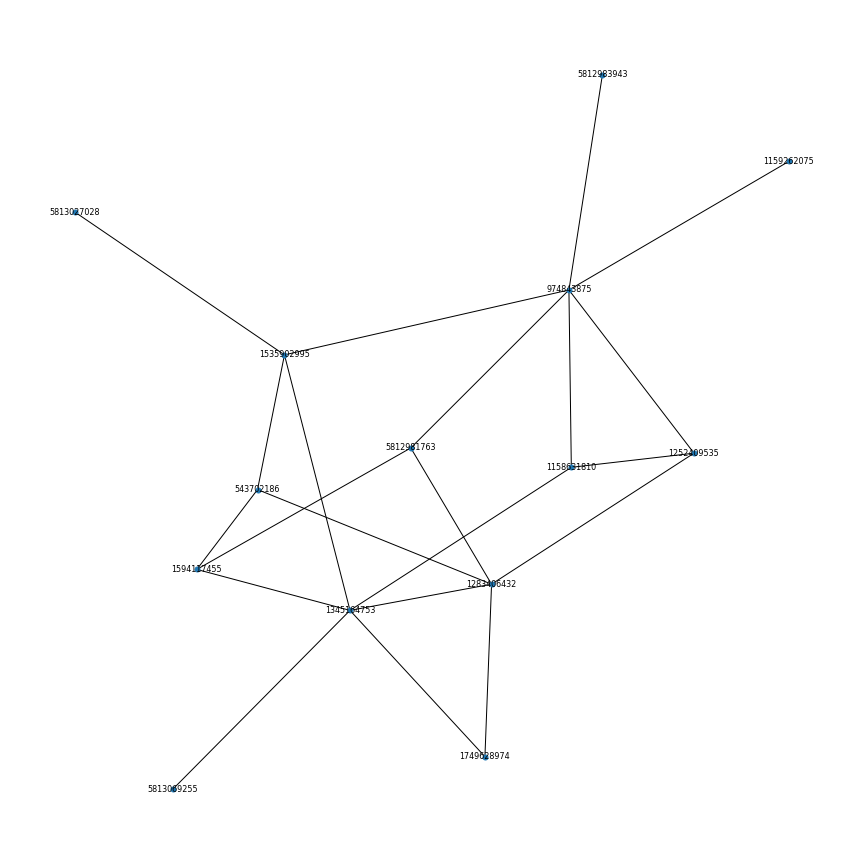

Supplement: Supplementary file 1 [file neurosci-03-00007-s001.zip › square_grid_windows/graph-ME(R)-1-1535902995.png]

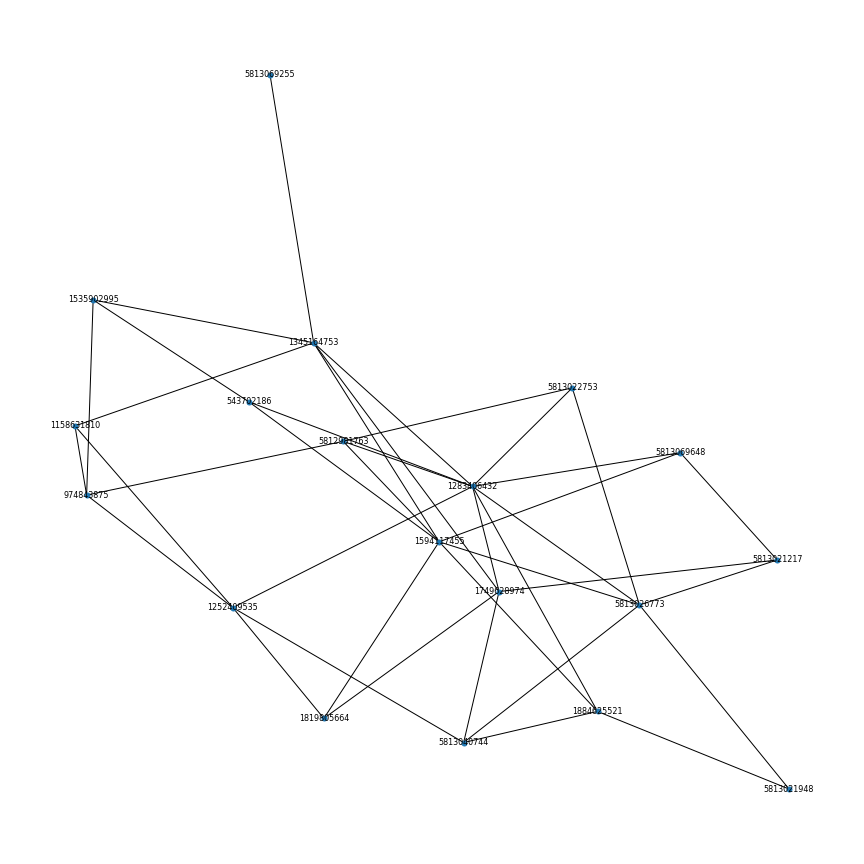

Supplement: Supplementary file 1 [file neurosci-03-00007-s001.zip › square_grid_windows/graph-ME(R)-1-1594117455.png]

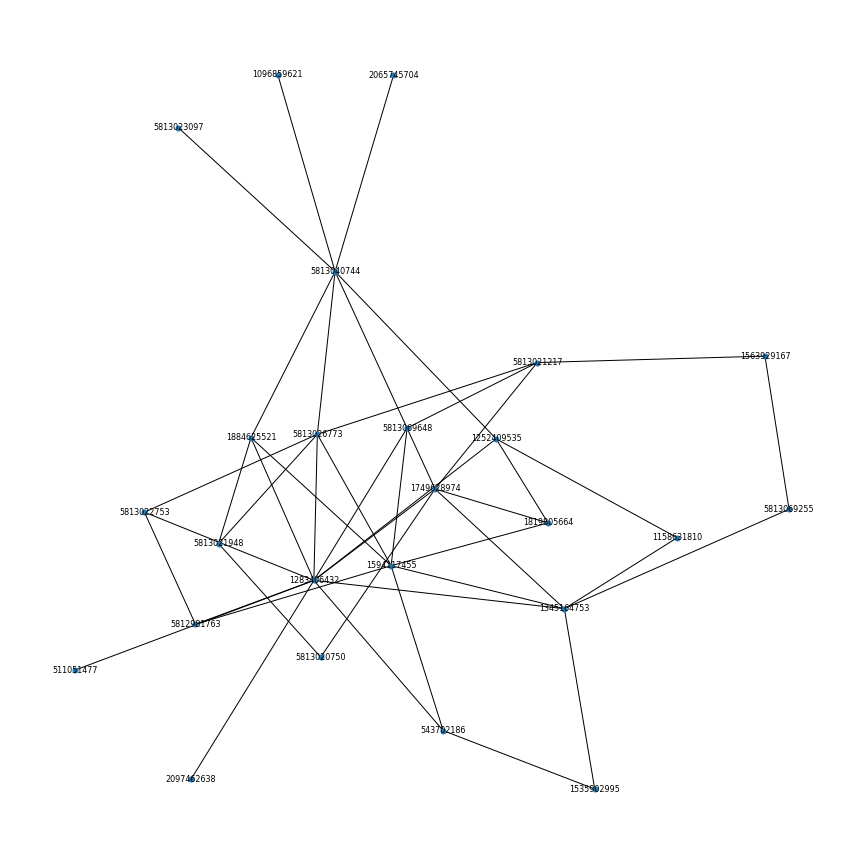

Supplement: Supplementary file 1 [file neurosci-03-00007-s001.zip › square_grid_windows/graph-ME(R)-1-1749628974.png]

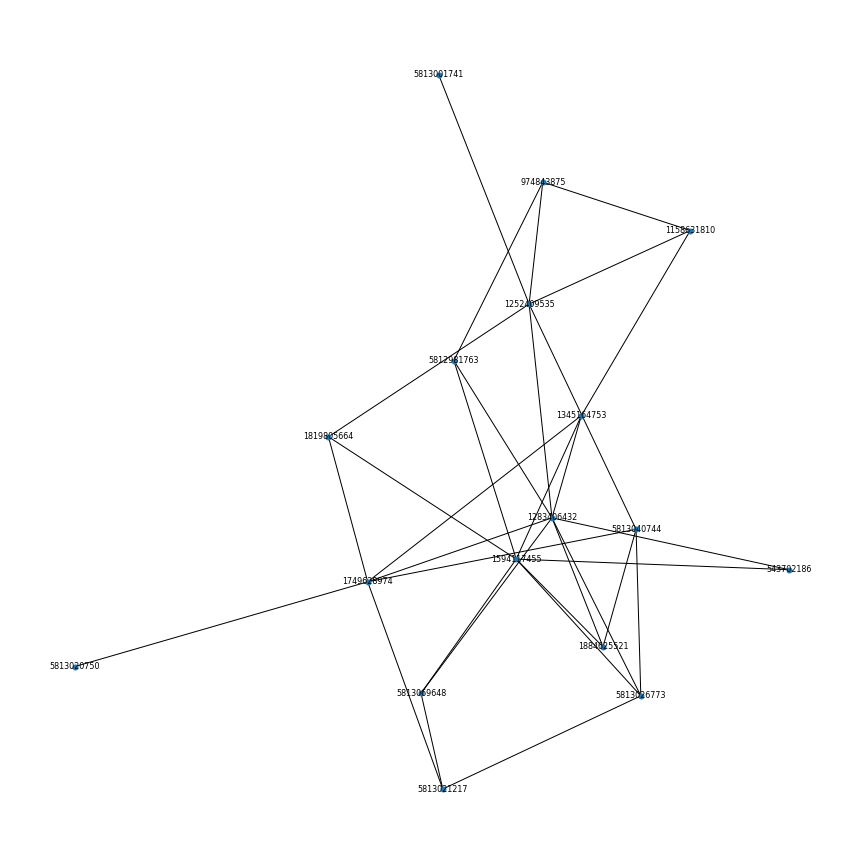

Supplement: Supplementary file 1 [file neurosci-03-00007-s001.zip › square_grid_windows/graph-ME(R)-1-1819805664.png]

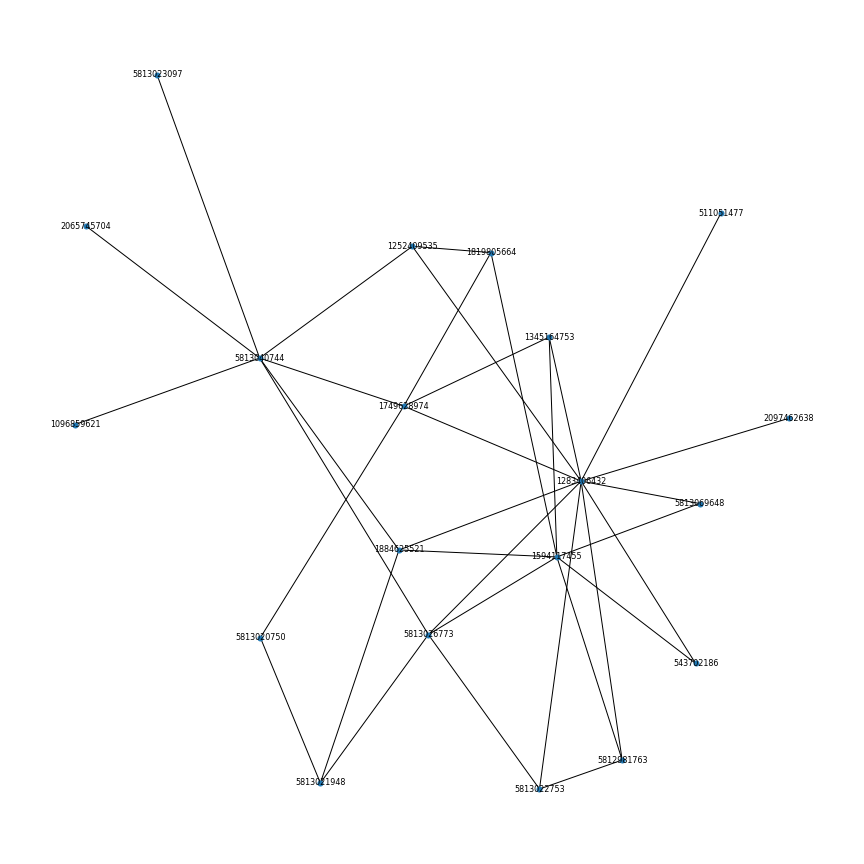

Supplement: Supplementary file 1 [file neurosci-03-00007-s001.zip › square_grid_windows/graph-ME(R)-1-1884625521.png]

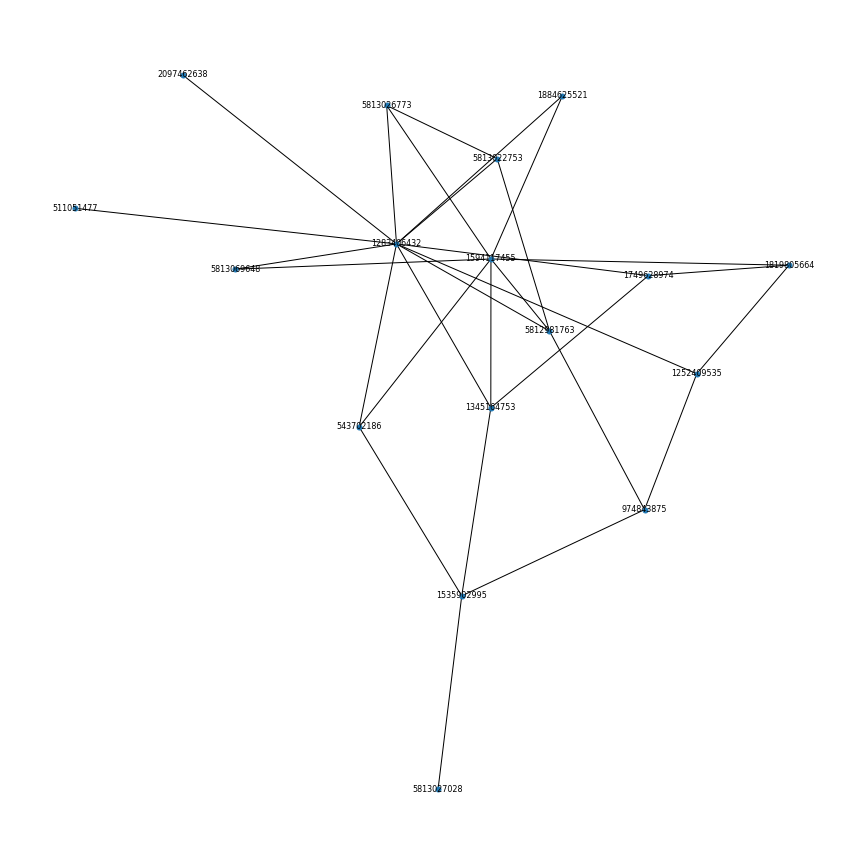

Supplement: Supplementary file 1 [file neurosci-03-00007-s001.zip › square_grid_windows/graph-ME(R)-1-543702186.png]

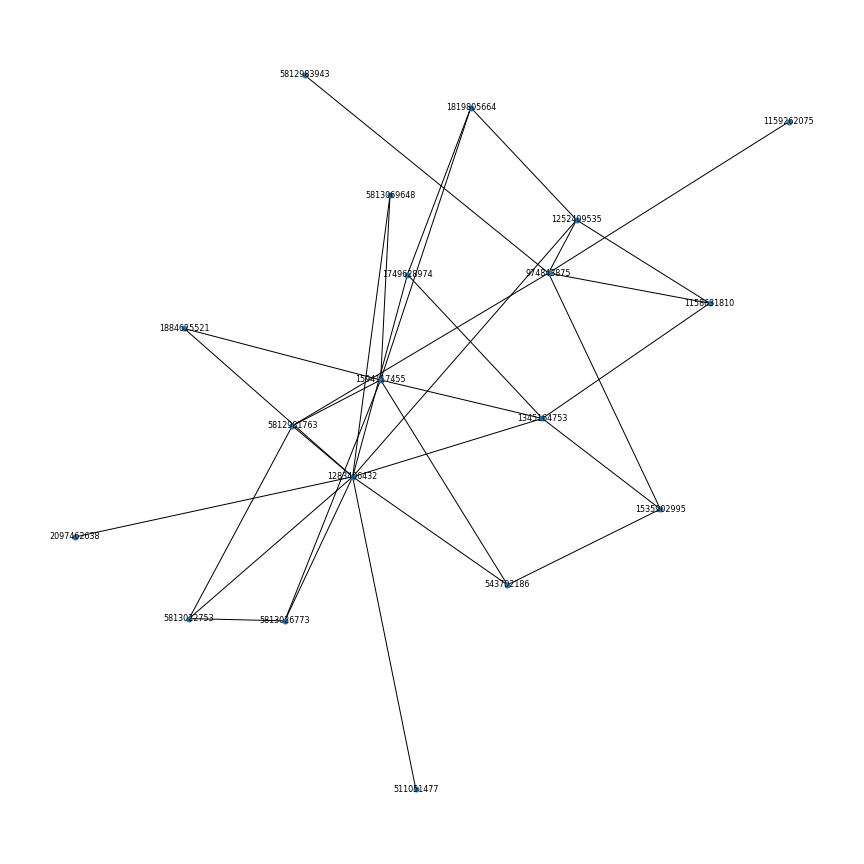

Supplement: Supplementary file 1 [file neurosci-03-00007-s001.zip › square_grid_windows/graph-ME(R)-1-5812981763.png]

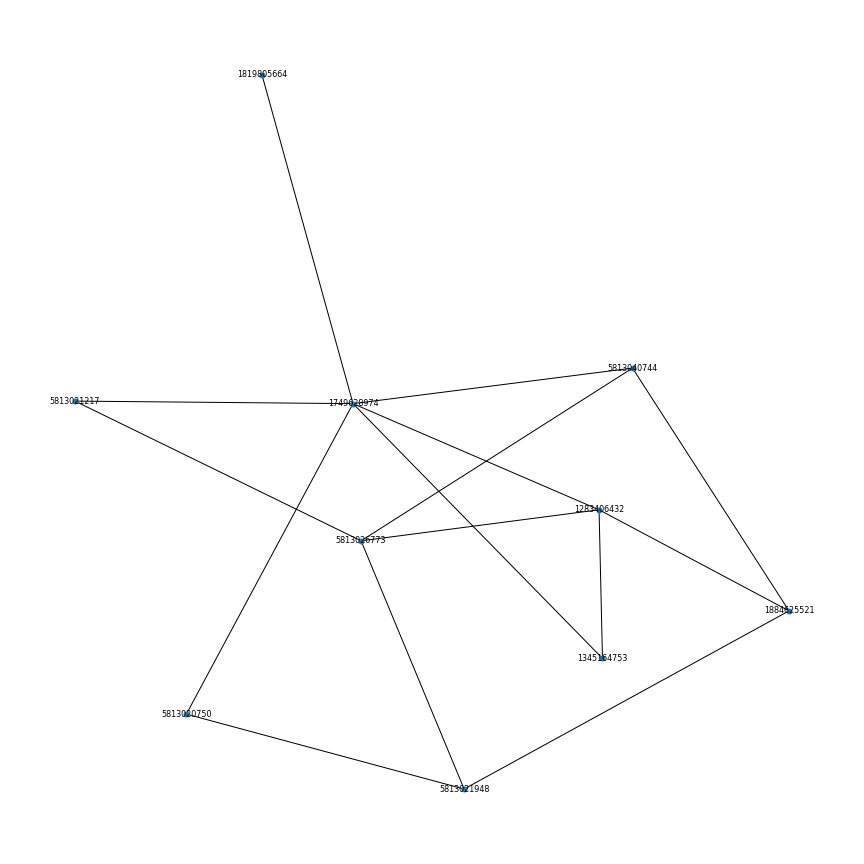

Supplement: Supplementary file 1 [file neurosci-03-00007-s001.zip › square_grid_windows/graph-ME(R)-1-5813020750.png]

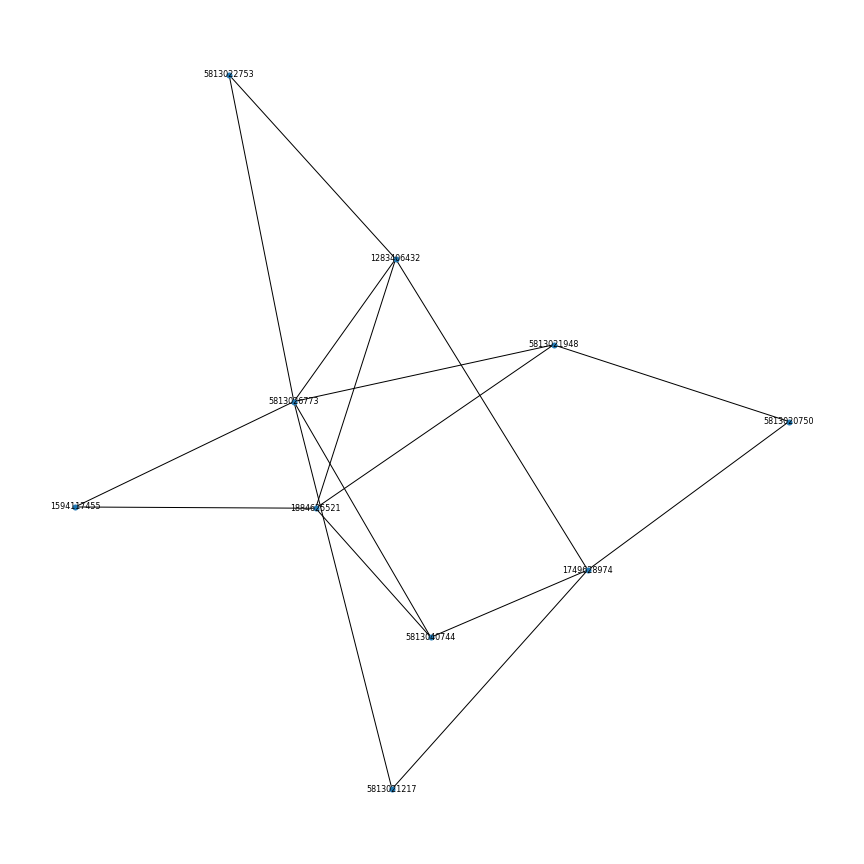

Supplement: Supplementary file 1 [file neurosci-03-00007-s001.zip › square_grid_windows/graph-ME(R)-1-5813021948.png]

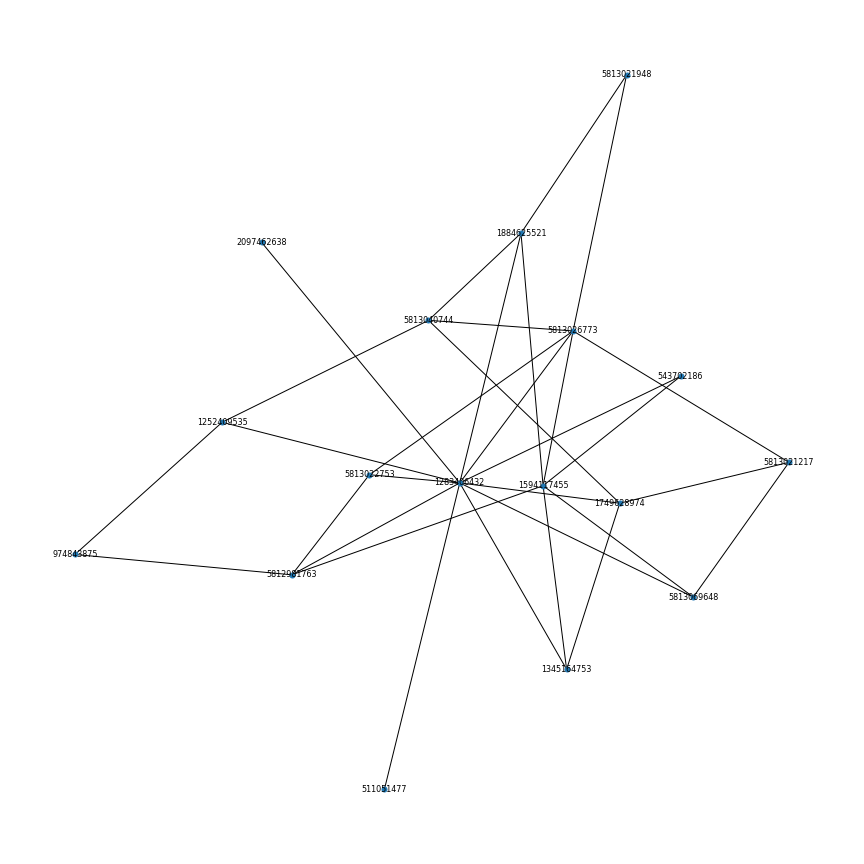

Supplement: Supplementary file 1 [file neurosci-03-00007-s001.zip › square_grid_windows/graph-ME(R)-1-5813022753.png]

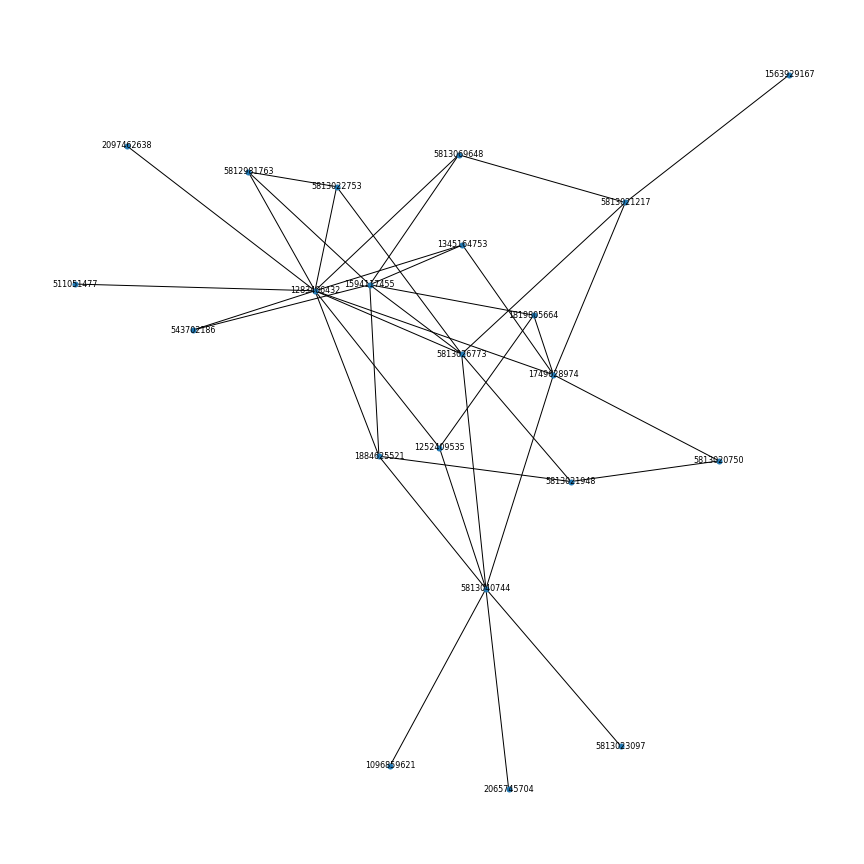

Supplement: Supplementary file 1 [file neurosci-03-00007-s001.zip › square_grid_windows/graph-ME(R)-1-5813026773.png]

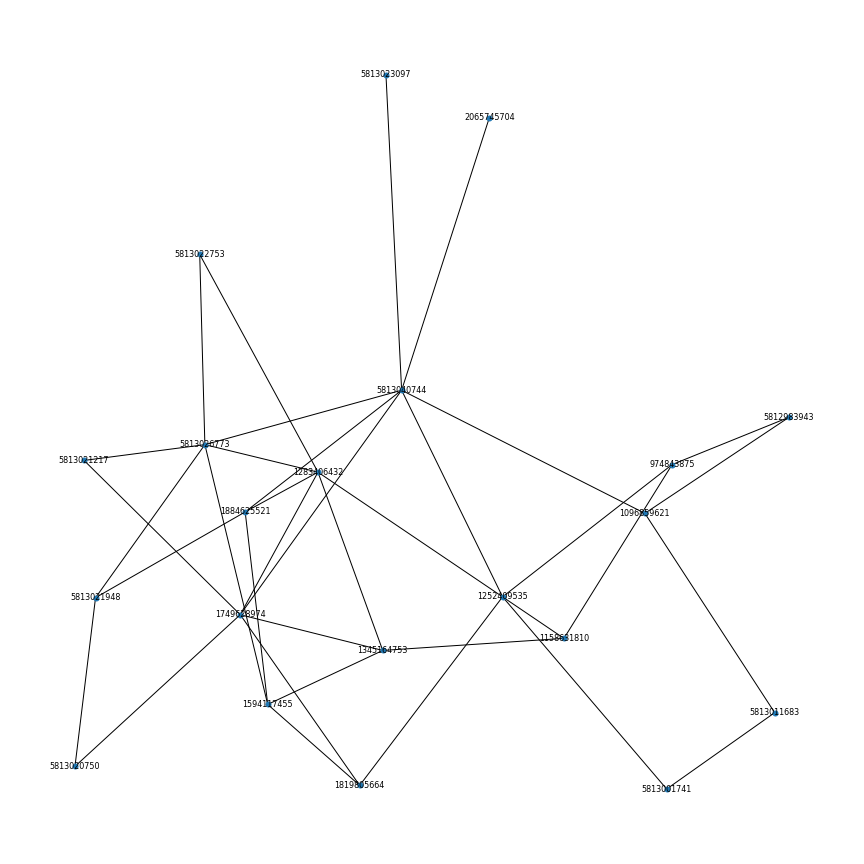

Supplement: Supplementary file 1 [file neurosci-03-00007-s001.zip › square_grid_windows/graph-ME(R)-1-5813040744.png]

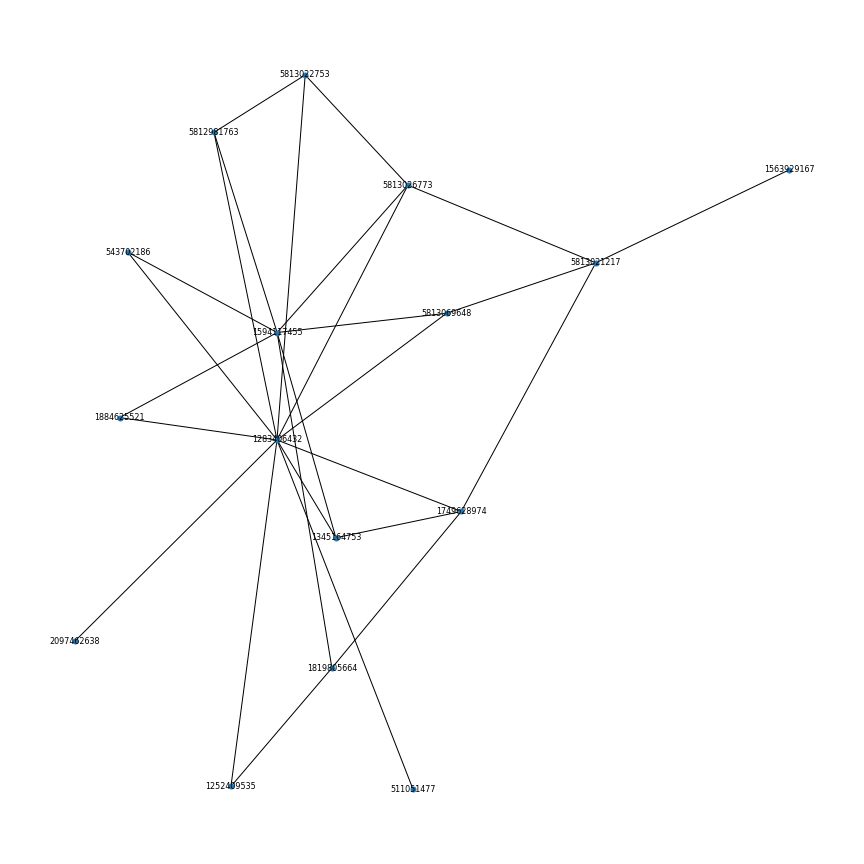

Supplement: Supplementary file 1 [file neurosci-03-00007-s001.zip › square_grid_windows/graph-ME(R)-1-5813069648.png]

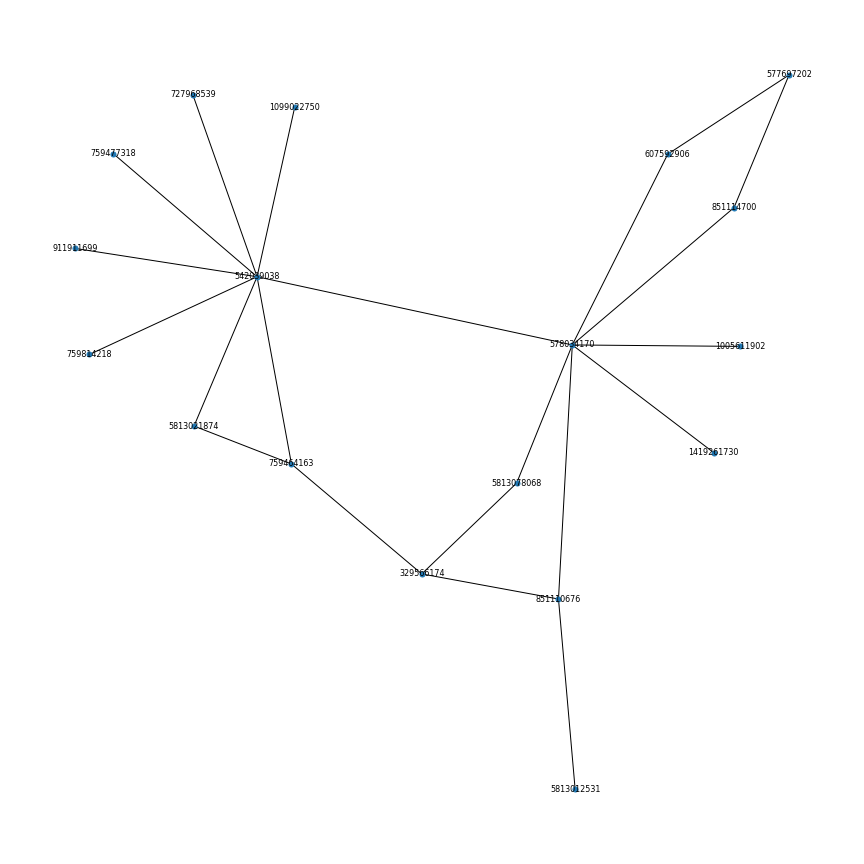

Supplement: Supplementary file 1 [file neurosci-03-00007-s001.zip › square_grid_windows/graph-PB-0-578034170.png]

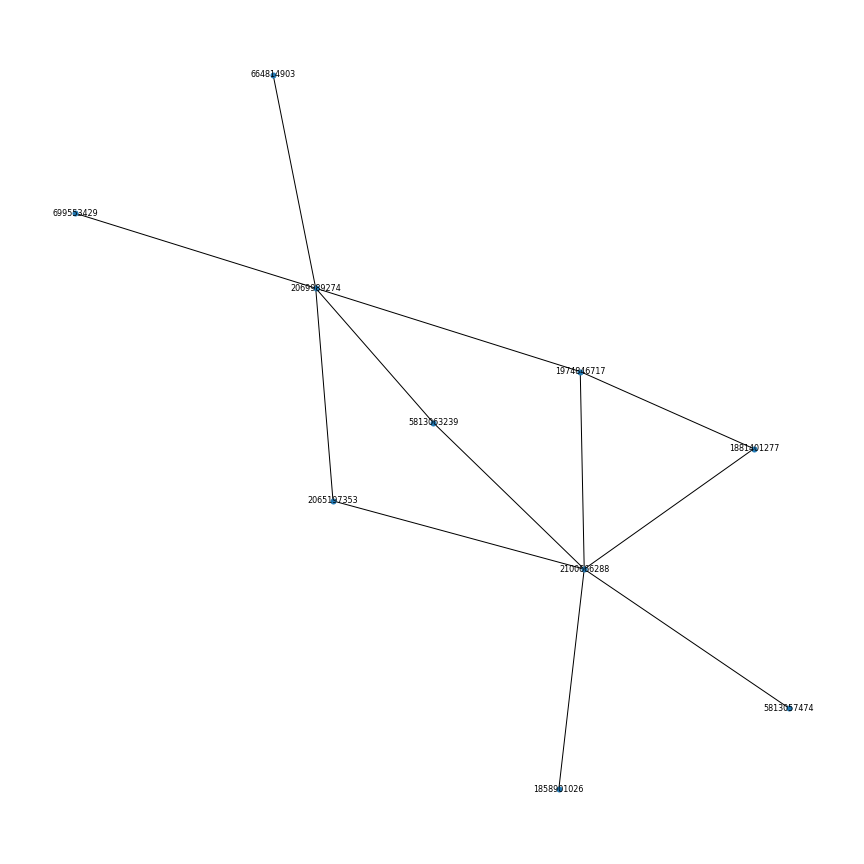

Supplement: Supplementary file 1 [file neurosci-03-00007-s001.zip › square_grid_windows/graph-SAD-1-2065197353.png]

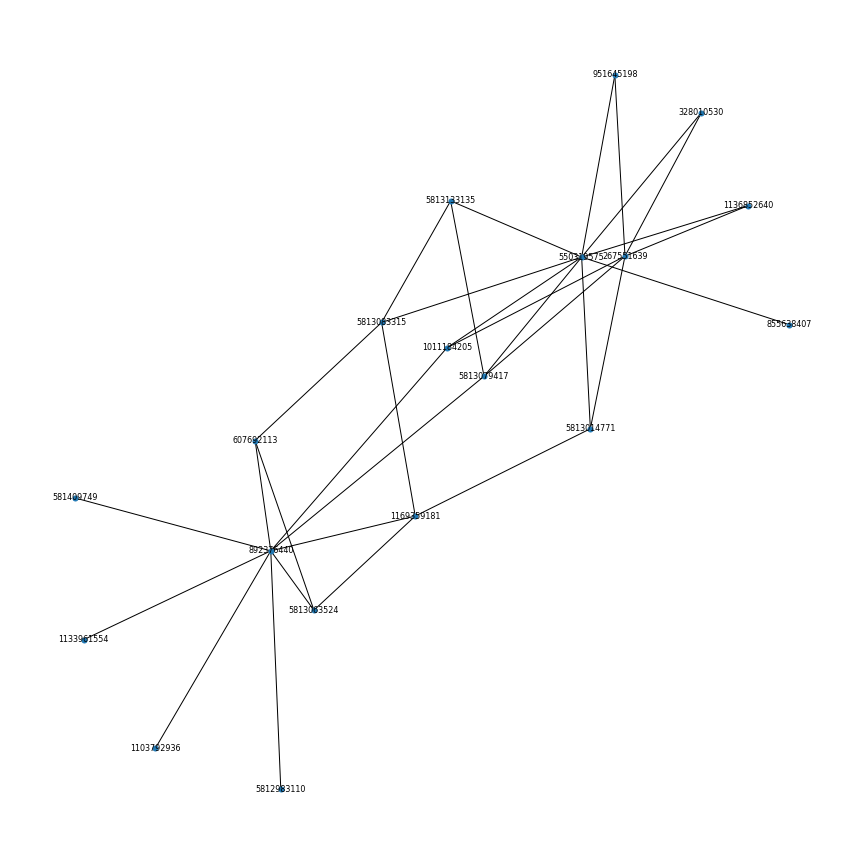

Supplement: Supplementary file 1 [file neurosci-03-00007-s001.zip › square_grid_windows/graph-SCL(L)-1-1011184205.png]

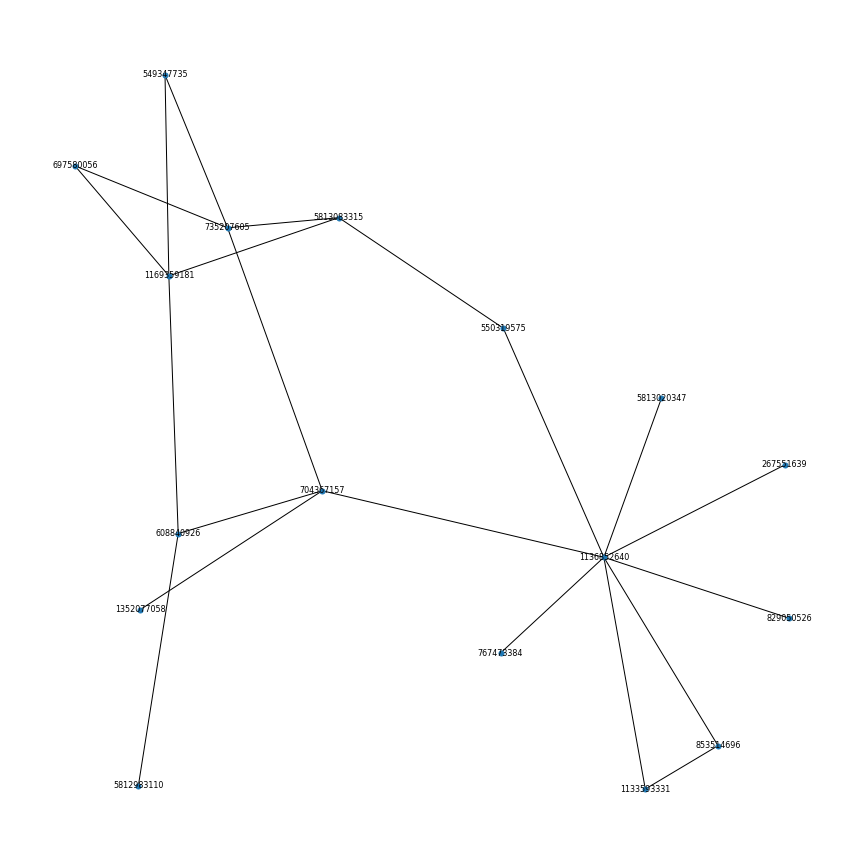

Supplement: Supplementary file 1 [file neurosci-03-00007-s001.zip › square_grid_windows/graph-SCL(L)-1-704367157.png]

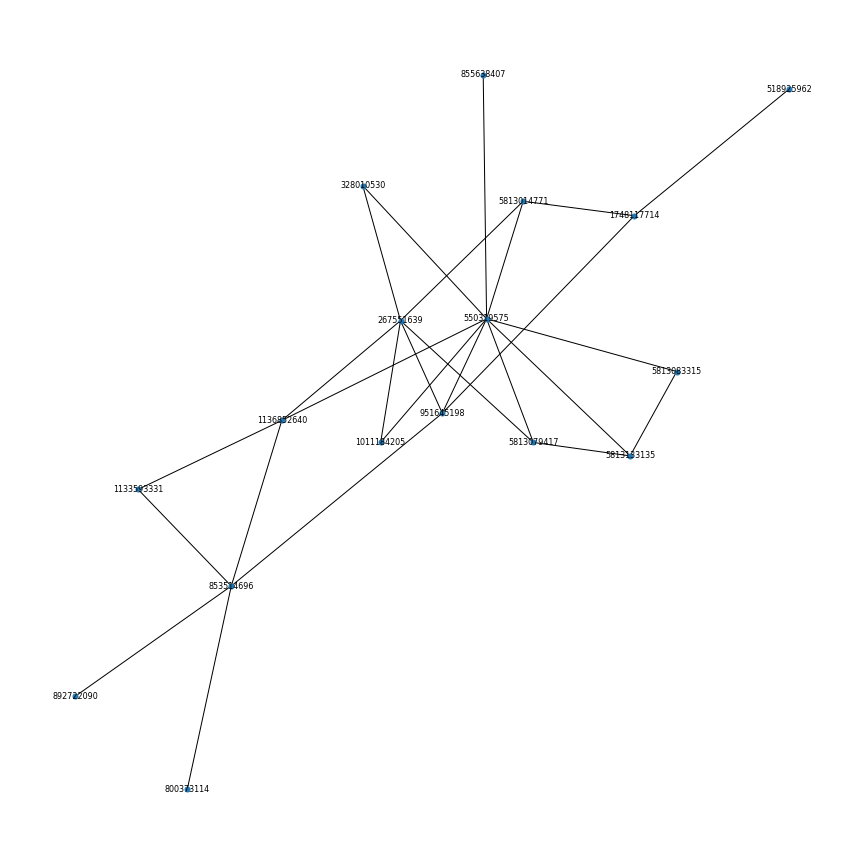

Supplement: Supplementary file 1 [file neurosci-03-00007-s001.zip › square_grid_windows/graph-SCL(L)-1-951645198.png]

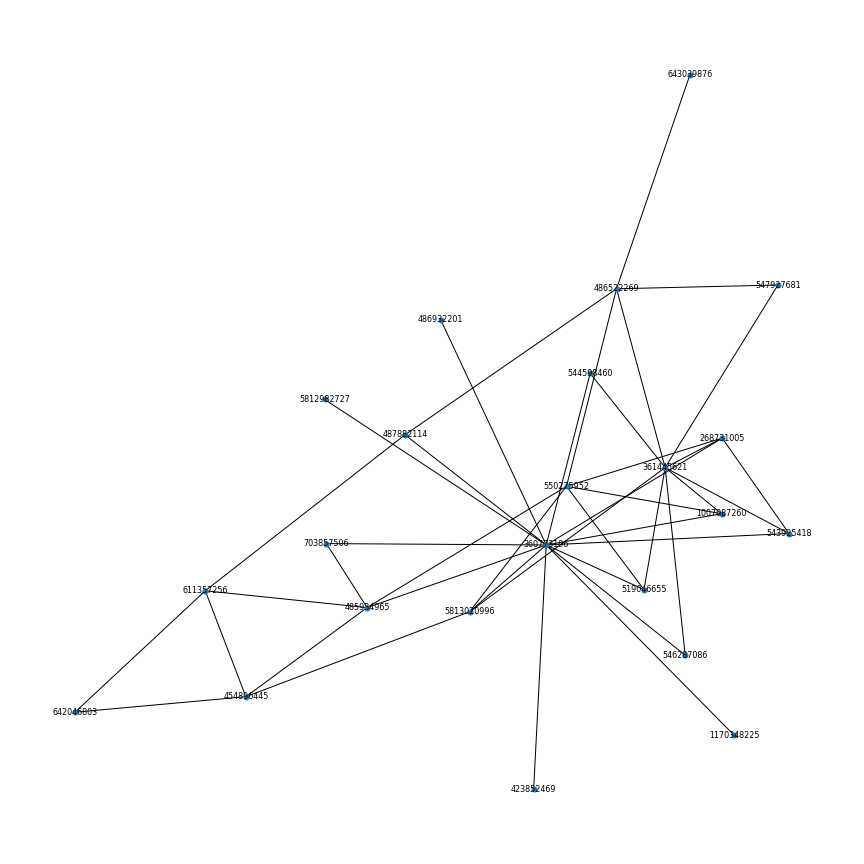

Supplement: Supplementary file 1 [file neurosci-03-00007-s001.zip › square_grid_windows/graph-SIP(L)-0-487882114.png]

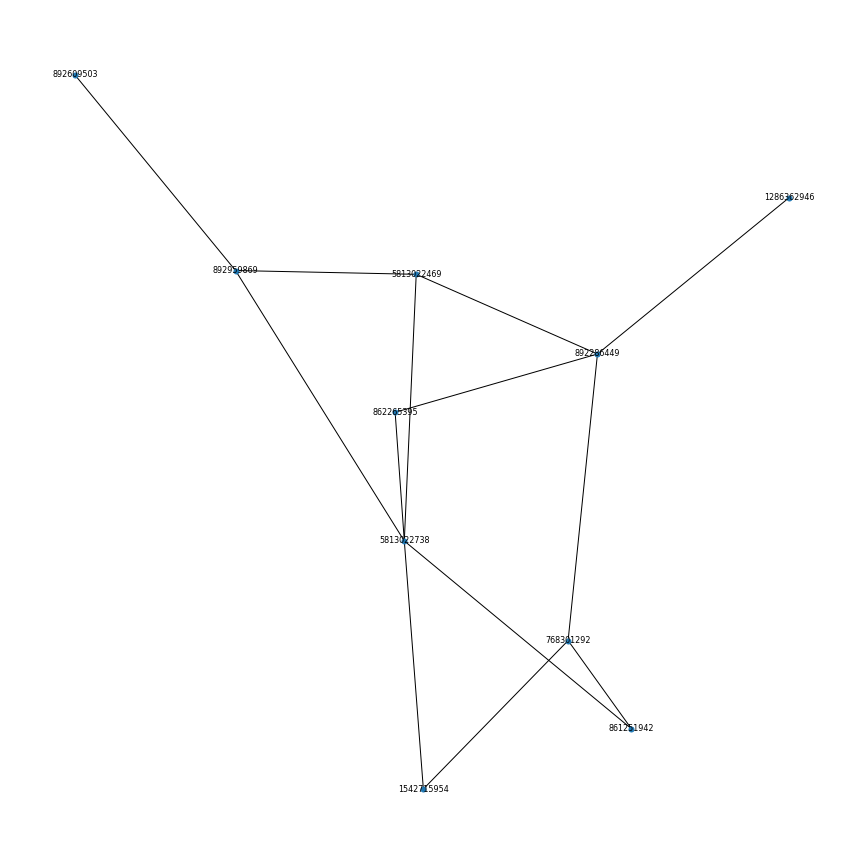

Supplement: Supplementary file 1 [file neurosci-03-00007-s001.zip › square_grid_windows/graph-SIP(L)-0-5813022469.png]

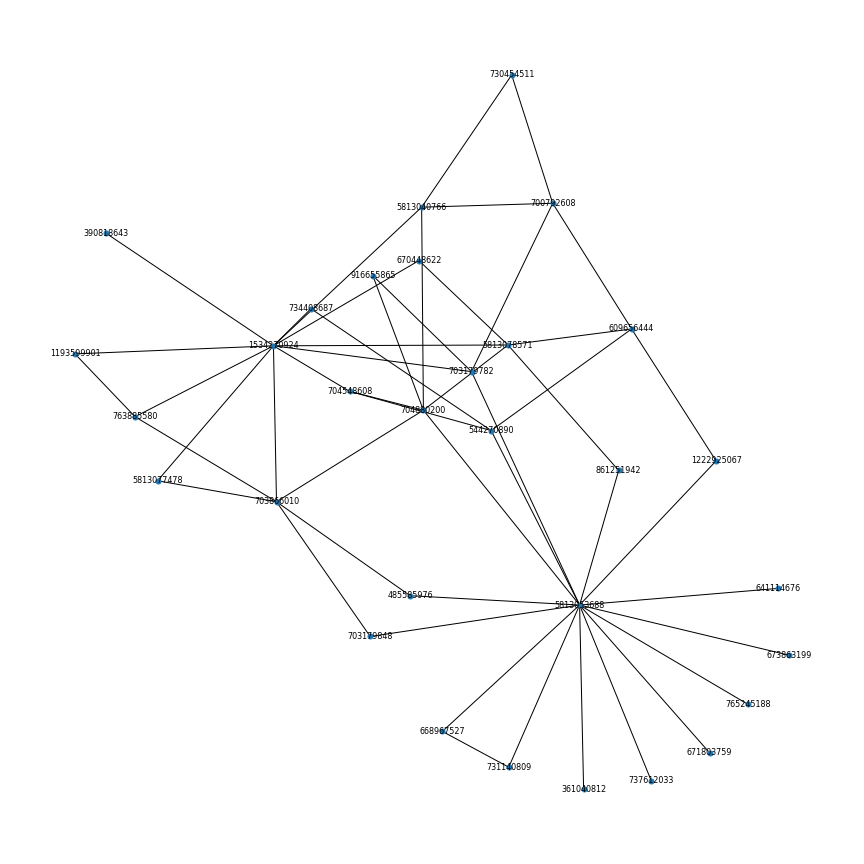

Supplement: Supplementary file 1 [file neurosci-03-00007-s001.zip › square_grid_windows/graph-SIP(L)-0-703179782.png]

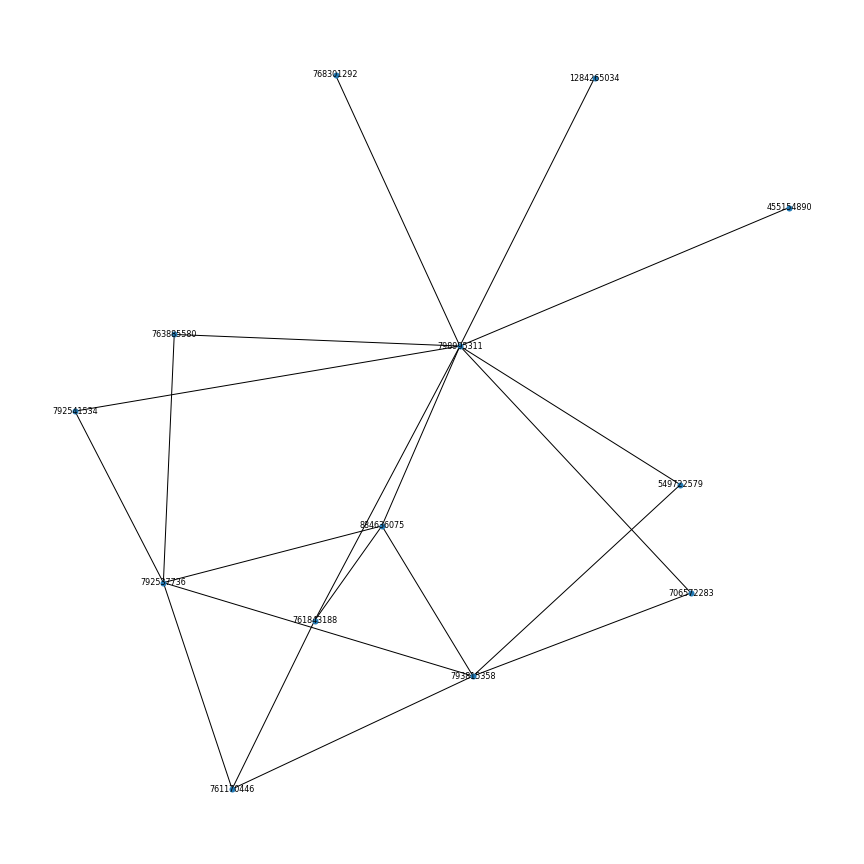

Supplement: Supplementary file 1 [file neurosci-03-00007-s001.zip › square_grid_windows/graph-SIP(L)-0-706572283.png]

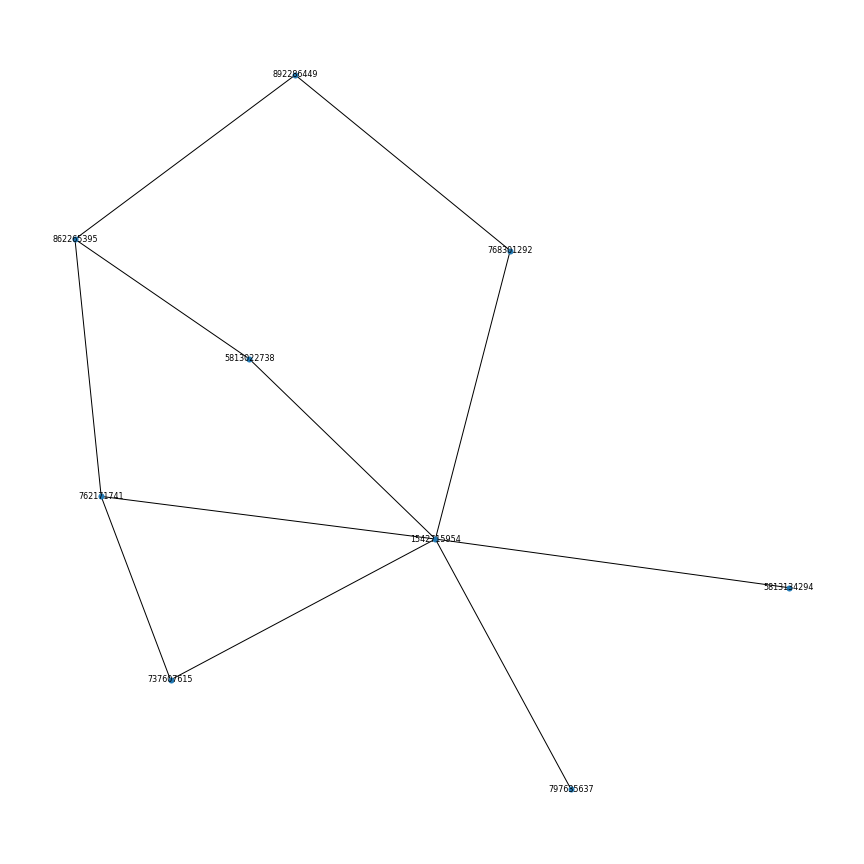

Supplement: Supplementary file 1 [file neurosci-03-00007-s001.zip › square_grid_windows/graph-SIP(L)-0-762171741.png]

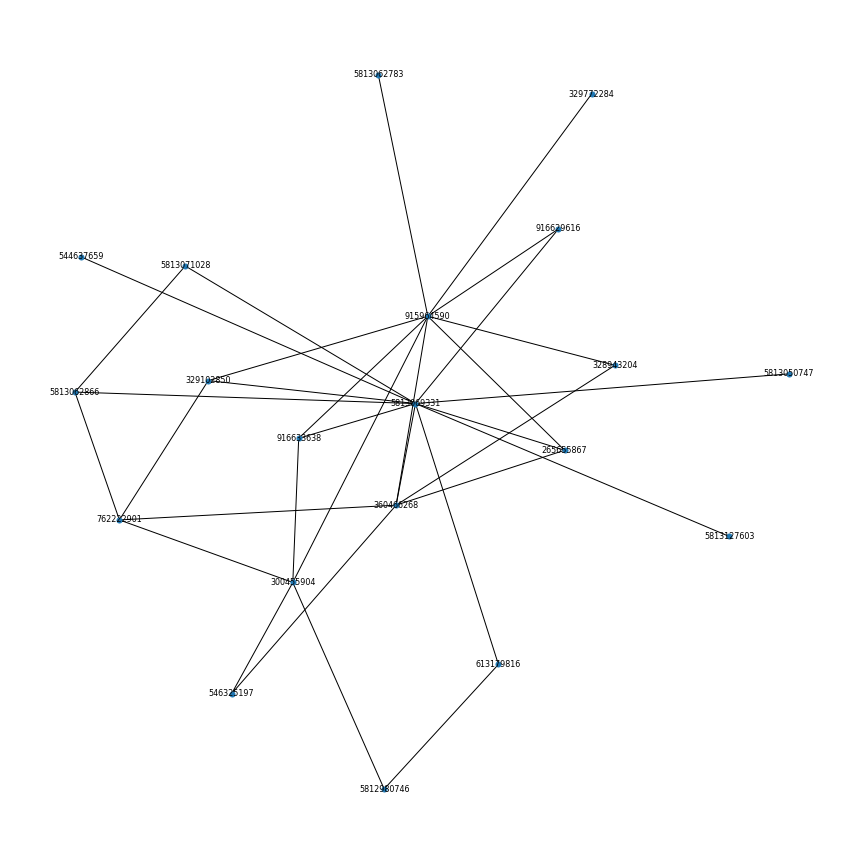

Supplement: Supplementary file 1 [file neurosci-03-00007-s001.zip › square_grid_windows/graph-SIP(L)-0-916633638.png]
